# Antagonistic Gene Activities Determine the Formation of Pattern Elements along the Mediolateral Axis of the *Arabidopsis* Fruit

**DOI:** 10.1371/journal.pgen.1003020

**Published:** 2012-11-01

**Authors:** Santiago González-Reig, Juan José Ripoll, Antonio Vera, Martin F. Yanofsky, Antonio Martínez-Laborda

**Affiliations:** 1 División de Genética, Universidad Miguel Hernández, San Juan de Alicante, Spain; 2 Division of Biological Sciences, University of California San Diego, La Jolla, California, United States of America; Peking University, China

## Abstract

The *Arabidopsis* fruit mainly consists of a mature ovary that shows three well defined territories that are pattern elements along the mediolateral axis: the replum, located at the medial plane of the flower, and the valve and the valve margin, both of lateral nature. *JAG*/*FIL* activity, which includes the combined functions of *JAGGED* (*JAG*), *FILAMENTOUS FLOWER* (*FIL*), and *YABBY3* (*YAB3*), contributes to the formation of the two lateral pattern elements, whereas the cooperating genes *BREVIPEDICELLUS* (*BP*) and *REPLUMLESS* (*RPL*) promote replum development. A recent model to explain pattern formation along the mediolateral axis hypothesizes that *JAG*/*FIL* activity and *BP*/*RPL* function as antagonistic lateral and medial factors, respectively, which tend to repress each other. In this work, we demonstrate the existence of mutual exclusion mechanisms between both kinds of factors, and how this determines the formation and size of the three territories. Medial factors autonomously constrain lateral factors so that they only express outside the replum, and lateral factors negatively regulate the medially expressed *BP* gene in a non-autonomous fashion to ensure correct replum development. We also have found that *ASYMMETRIC LEAVES1* (*AS1*), previously shown to repress *BP* both in leaves and ovaries, collaborates with *JAG*/*FIL* activity, preventing its repression by *BP* and showing synergistic interactions with *JAG*/*FIL* activity genes. Therefore *AS* gene function (the function of the interacting genes *AS1* and *AS2*) has been incorporated in the model as a new lateral factor. Our model of antagonistic factors provides explanation for mutant fruit phenotypes in *Arabidopsis* and also may help to understand natural variation of fruit shape in Brassicaceae and other species, since subtle changes in gene expression may cause conspicuous changes in the size of the different tissue types.

## Author Summary

There are three main pattern elements in the mediolateral axis of the *Arabidopsis* fruit. Two of them, the valves and the valve margins, are placed in lateral positions, while the third, called replum, is located in the medial plane of the flower. The replum expresses meristematic genes (medial factors) that specify its development, whereas the function of genes that work in leaves (lateral factors) determines the development of valves and valve margins. Consequently, medial and lateral pattern elements of fruits apparently mimic the antagonistic relationships between meristem and leaves. According to this, we propose a model for mediolateral patterning of fruits whereby the mutual opposing activities of medial and lateral factors drive the formation of replum, valves, and valve margins. We conclude that medial factors function in an autonomous fashion to prevent the expression of lateral factors in the replum, and that lateral factors repress medial factors by a non-autonomous mechanism to allow normal replum development. Our model provides explanation for changes in fruit shape in Brassicaceae and related organisms either by mutation within a species or by natural variation among different species.

## Introduction

**Figure 1:**
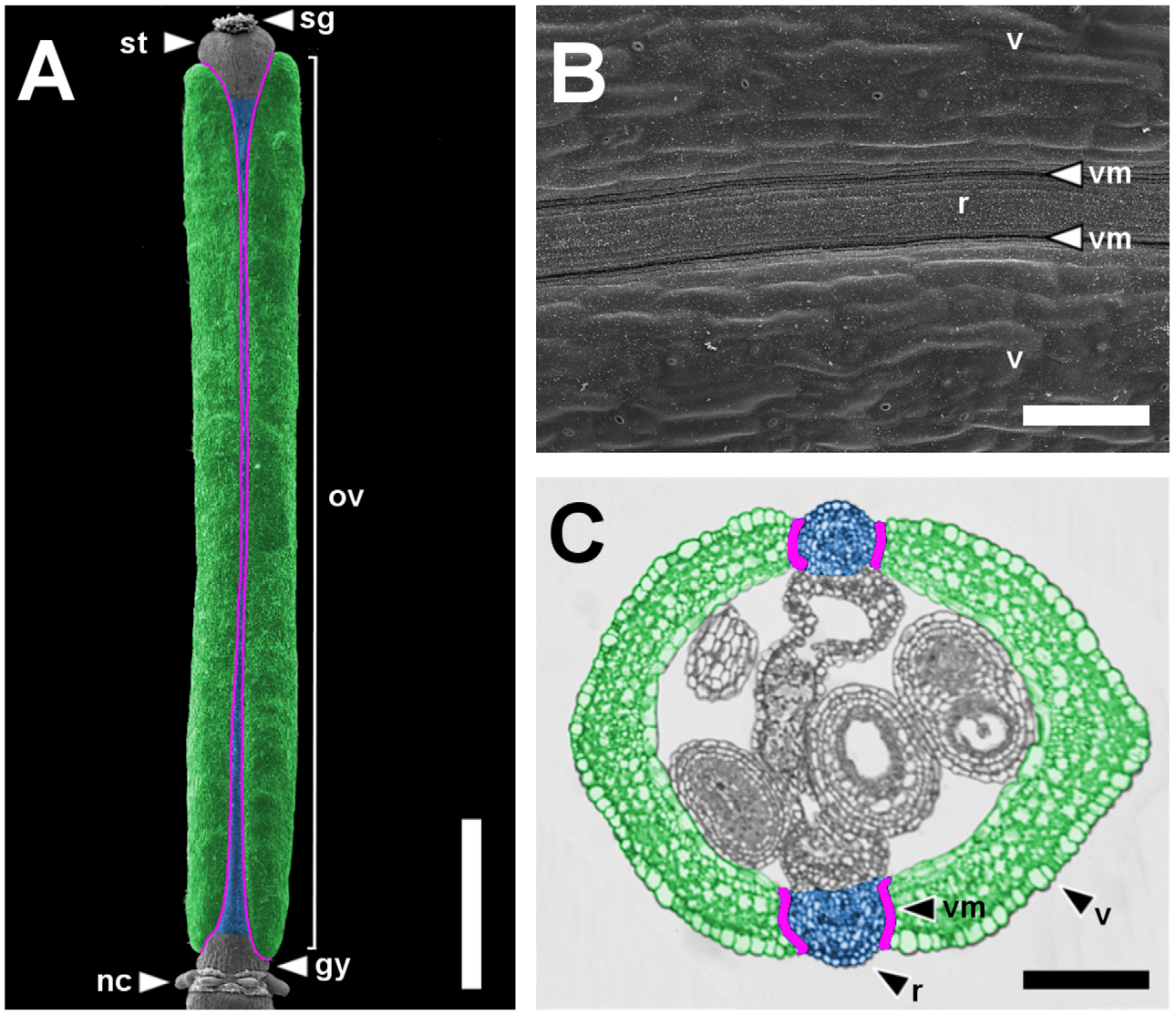
Main pattern elements along the mediolateral axis of the *Arabidopsis* wild-type fruit. Scanning Electron Microscope (SEM) micrograph of a stage 17 fruit artificially colored to highlight the main pattern elements along the mediolateral axis: valves in green, replum in blue and valve margins in purple (A) and a higher magnification of the same fruit (B). Cross-section of a fruit at the level of the ovary with the same color code as in A (C). gy, gynophore; nc, nectaries; ov, ovary; r, replum; sg, stigma; st, style; v, valve; vm, valve margin. Scale bars: 1 mm (A), 100 µm (B, C).

The fruit, a pivotal structure in angiosperms, is the specialized plant organ that develops from the gynoecium after fertilization of the ovules. The very term angiosperm comes from the Greek and means “seeds enclosed in a vessel” (*angion*, vessel, and *sperma*, seed), describing the main functions of this organ: seed protection and dispersal. Our present knowledge on fruit development principally derives from research in the crucifer *Arabidopsis thaliana*, *Arabidopsis* hereafter [1]–[7]. All the tissues of the *Arabidopsis* fruit are already present in the bicarpelate pistil, whose development is initiated as a group of cells that form a dome-shaped primordium. Subsequently, polarity is determined along the main axes of symmetry giving rise to pattern elements with specific tissue types. Thus, for instance, along the apical-basal axis both pistils and fruits show, from bottom to top, the basal gynophore, the ovary, the style and the apical stigma (Figure 1A).

The dehiscent fruit of *Arabidopsis* is essentially an expanded ovary encompassing the seeds [8], and consists of three different territories that constitute the pattern elements along the mediolateral axis. The replum, located at the medial plane of the flower, is a narrow structure that separates two lateral valves. At the valve-replum boundary, the valve margin, another lateral tissue, comprises a few rows of small and rounded cells (Figure 1A–1C). Ripening of the fruit involves the formation of a dehiscence zone in the valve margin and the consequent detachment of the valves from the replum that precedes seed dispersal [9].

The MADS-box gene *FRUITFULL* (*FUL*) [10] and the homeobox gene *REPLUMLESS* (*RPL*, aka *BELLRINGER*, *PENNYWISE*, *LARSON* and *VAAMANA*) [11]–[14] are expressed within the valve and replum tissues, respectively. *RPL* and *FUL*, in their corresponding domains of activity, prevent the ectopic expression of the valve margin identity genes *SHATTERPROOF1* and *2* (*SHP1*, *SHP2*) [15], *INDEHISCENT* (*IND*) [16] and *ALCATRAZ* (*ALC*) [17]. This regulation ensures the correct formation of valves and replum territories and limits the expression of the valve margin identity genes to the valve-replum boundaries. Thus, in fruits completely lacking both *FUL* and *RPL* activities, valves and replum epidermal cells acquire valve margin identity as a consequence of the ectopic expression of valve margin identity genes [13], [16], [18].

Early in pistil development, medial tissues form two internal ridges that fuse to form the septum and the placenta, suggesting that presumptive repla have meristematic properties [5], [19], [20]. Accordingly, they exhibit expression of meristem genes, as *RPL* [13] and the class I *KNOTTED1*-like homeobox (KNOX) genes *BREVIPEDICELLUS* (*BP*, aka *KNAT1*) [21]–[24] and *SHOOT MERISTEMLESS* (*STM*) [25]. Different from the replum, valves show a more leaf-like nature, and consequently, they express genes with crucial roles in leaf development, as the YABBY1 (YAB1) group genes *FILAMENTOUS FLOWER* (*FIL*) and *YABBY3* (*YAB3*) [26]–[30], *JAGGED* (*JAG*), which codes for a transcription factor with a single C2H2 zinc-finger domain [31], [32], and the MYB transcription factor-encoding gene *ASYMMETRIC LEAVES1* (*AS1*) [33], [34]. *FIL*, *YAB3* and *JAG* positively regulate the expression of *FUL* and valve margin identity genes, so that the cooperating activities of these three genes in ovaries have been called *JAG*/*FIL* activity. Valve margin identity genes are activated in places close to the presumptive replum where the levels of this activity are low, whereas higher levels activate *FUL* expression in valves [35].

Replum and valves apparently mirror the antagonistic relationships between meristem and leaves [19], [20]. Thus, *AS1* prevents the ectopic expression of *BP* in valves while *RPL* impedes that of *JAG*/*FIL* activity genes in the replum [19], [35]. Based on this antagonism, a model has been proposed to account for mediolateral patterning of the ovary, which puts forward that the different tissue types along the mediolateral axis are determined by the opposing activities of two antagonistic factors: valve factors that basically are the genes involved in the *JAG*/*FIL* activity, and replum factors that are composed by *BP* and *RPL* [19], [36], whose products dimerize to migrate into the nucleus [11], [14], [37]–[42]. In accordance to the model, replum and valves would form in territories with high activity of replum and valve factors, respectively, whereas the valve margin would develop in a narrow ridge in which both activities would show low levels and overlap [19].

Nevertheless, recent research has shown that valve and replum factor activities do not overlap, since *BP* and *RPL* are not active in the valve margin [36], [43] (our unpublished results). Therefore, *BP* and *RPL* will be hereafter referred to as replum or medial factors, whereas genes involved in *JAG*/*FIL* activity (hereafter referred as *JAG*/*FIL* activity genes) will be called lateral (valve and valve margin) factors. In this report, we demonstrate that, indeed, both medial and lateral factors are mutually antagonistic, as they repress each other. We have observed that lateral factors negatively regulate in a non-autonomous fashion *BP*, thus restricting the size of the medial region, an essential condition for proper replum development, whereas medial factors limit in an autonomous way the expression of *JAG*/*FIL* activity genes, whose products only are detected outside the replum. Furthermore, we have also found that *AS1* collaborates with lateral factors by preventing downregulation of *JAG*/*FIL* activity genes by the ectopic expression of *BP* in lateral regions. Here, we propose a non-overlapping model whereby the opposing activities of medial and lateral factors determine the specification and size of pattern elements along the mediolateral axis of the *Arabidopsis* fruit. In accordance with this model, an increase in the expression of medial factors and a decrease in lateral factor activities lead to the overproduction of medial tissues along with a large reduction in the size of the lateral domains.

## Results

### *BP* is involved in the replum defects of mutants with impaired *JAG*/*FIL* activity

**Figure 2:**
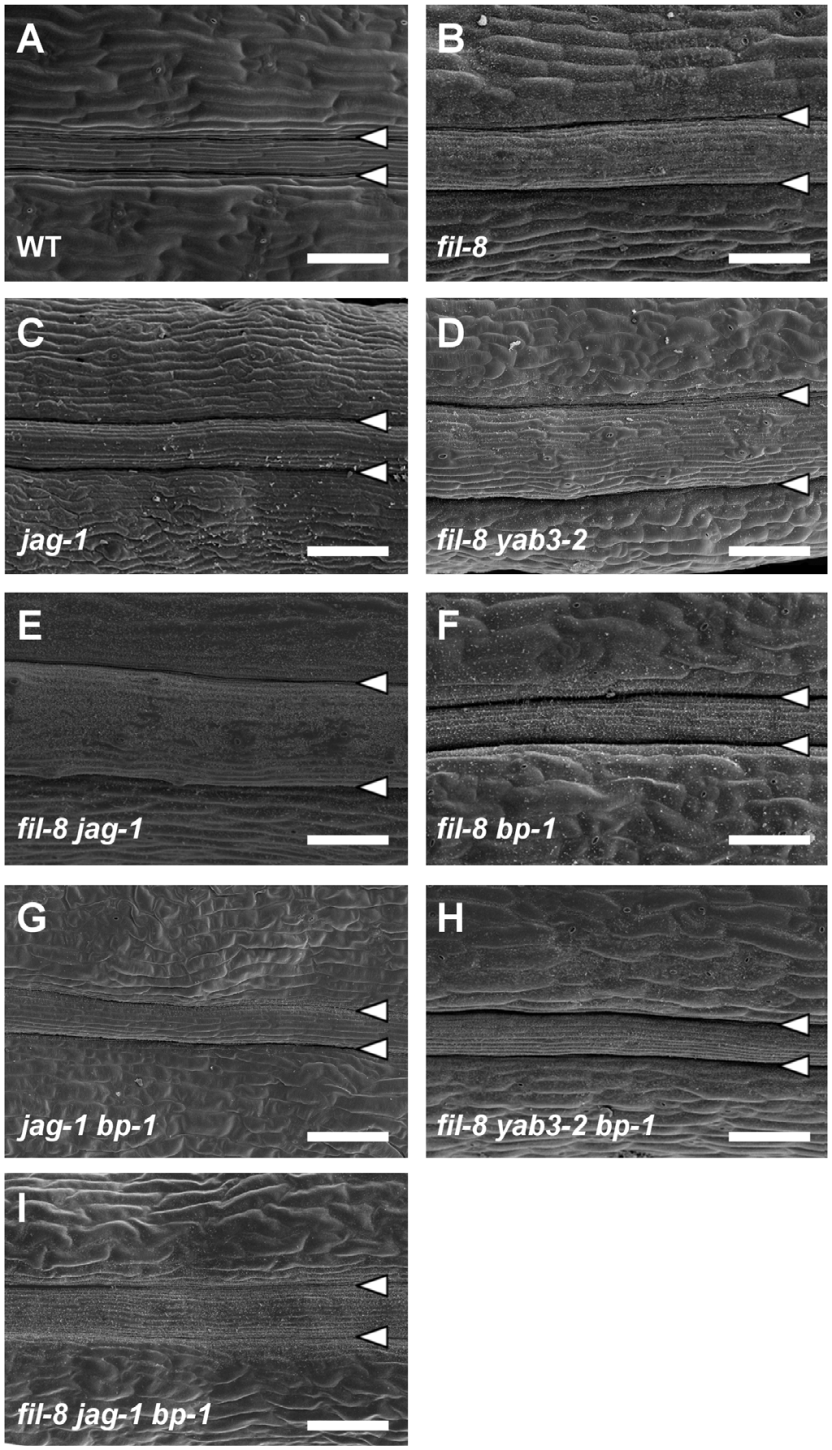
Abnormal repla in mutants affected in *JAG*/*FIL* activity. (A–E) SEM micrographs of stage 17 fruits show enlarged repla in single and multiple mutants affected in *JAG*/*FIL* activity. The *fil-8* (B) and *jag-1* (C) single mutants display large repla respect to the wild type (A). The *fil-8 yab3-2* (D) and *fil-8 jag-1* (E) double mutants exhibit more severe phenotypes. (F–I) SEM micrographs of stage 17 fruits showing the rescue of mutant phenotypes by *bp-1* in *fil-8 bp-1* (F), *jag-1 bp-1* (G), *fil-8 yab3-2 bp-1* (H) and *fil-8 jag-1 bp-1* (I) plants. Arrowheads indicate the positions of the valve margins. Scale bars: 100 µm.

We have previously demonstrated that the MYB transcription factor AS1 regulates patterning along the mediolateral axis of the *Arabidopsis* fruit. When compared to wild type, in *as1* fruit, the replum contains more epidermal cells, increasing its width. This phenotype is accompanied with a reduction in the final size of the valves as the valve epidermal layer contains fewer cells [19]. We previously found that the *as1* fruit phenotype was largely associated with the misregulation of *BP*, because: 1) *35S::BP* had the same fruit alterations as seen in *as1* plants, 2) *BP* was ectopically expressed in lateral regions of *as1* pistils and 3) in *as1 bp* fruits, replum and valves almost completely recovered the wild-type size [19]. However, the increase in the number of replum cells is not the only alteration observed in *as1* (or *35S::BP*) repla. Whereas in the wild-type pistils the replum contains long and narrow cells and no stomata structures form (Figure 1B and Figure 2A), a closer inspection of altered repla in *as1* and *35S::BP* plants revealed, on the contrary, the presence of extra-large cells, as well as a few interspersed stomata (Figure S1A, S1B). These observations indicate that the negative regulation of *BP* by *AS1* is not only essential in regulating the size of pattern elements along the mediolateral axis, but also for the correct specification of replum identity.

As mentioned in the introduction, *JAG*/*FIL* activity genes [35] have been postulated to be the valve functions (that we refer in this work as lateral factors) patterning the mediolateral axis of the fruit in *Arabidopsis*. Consequently, similar to *as1* mutants, a decrease in this activity drastically affects the valves [7], [19], [35]. Furthermore, according to our current model, reduced levels of *JAG*/*FIL* activity should not only cause a reduction in the size of the valve territory, but also a mutant replum phenotype consisting in increased width [7], [19]. Fitting with this hypothesis, we observed that fruits in *jag* and *fil* plants, besides their defects in lateral regions [35], clearly exhibited oversized repla (Figure 2B, 2C). Moreover, a close inspection of the replum surface by SEM revealed the presence of stomata in both *fil* and *jag* repla (Figure S1C, S1D). These abnormalities were even more dramatic when the *JAG*/*FIL* activity was further reduced, as for example in *fil yab3* or *fil jag* backgrounds (Figure 2D, 2E and Figure S1E, S1F).

Because of the similarities between these defects and the ones described before for *as1* or *35S::BP* repla [19], we investigated whether the lack of *BP* was capable of rescuing the fruit phenotypes of mutants affected in the *JAG*/*FIL* activity. Indeed, *fil bp*, *jag bp*, *fil yab3 bp* and *fil jag bp* fruits showed narrow repla and contained no replum stomata (Figure 2F–2I). These observations suggest that *JAG*/*FIL* activity regulates the expression of *BP* in the *Arabidopsis* fruit and that misregulation of *BP* is essential to produce the repla defects seen in mutants affected in this activity.

### *JAG*/*FIL* activity negatively regulates *BP* expression in fruits

**Figure 3:**
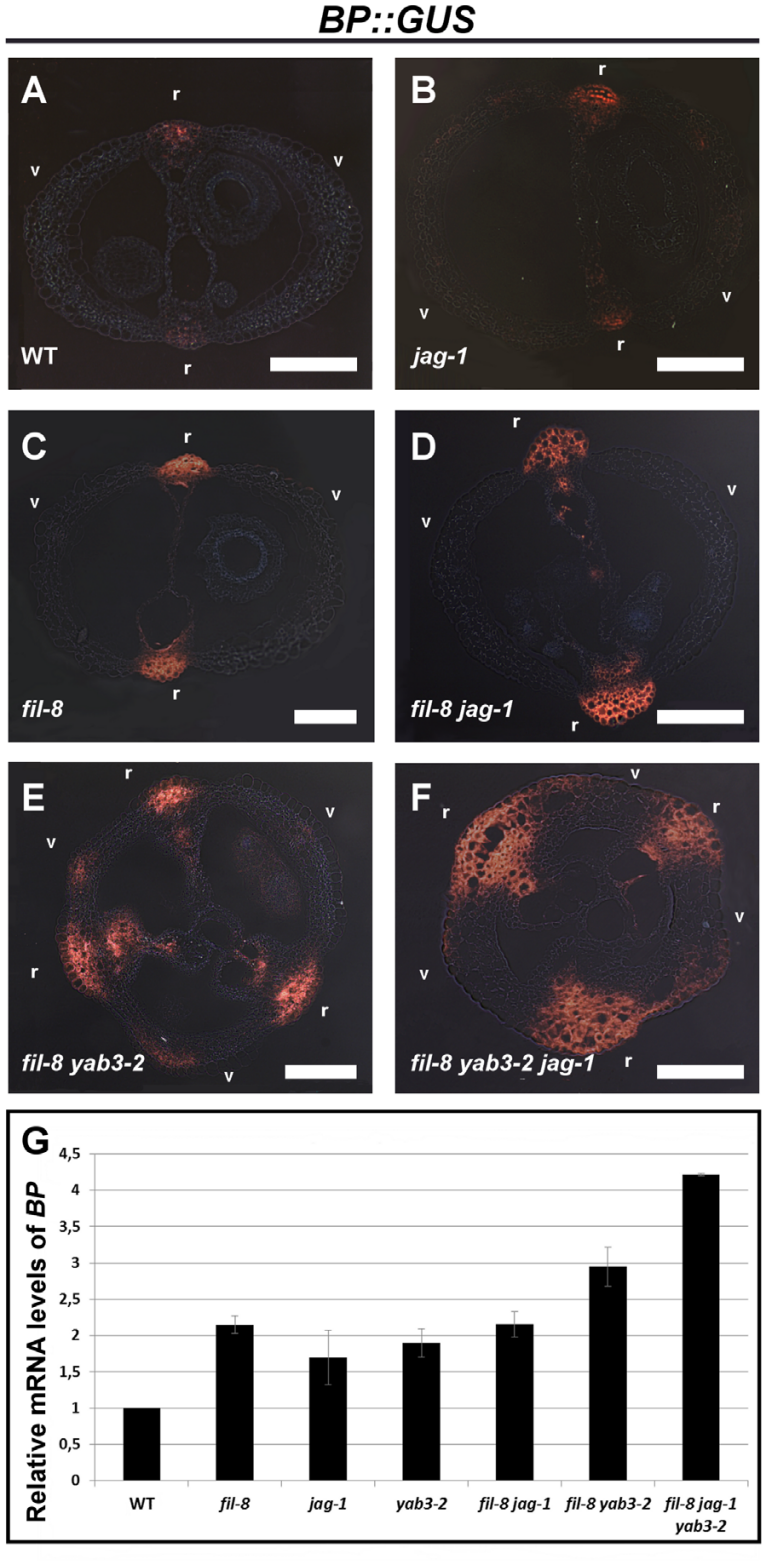
The Expression of *BP* increases in mutants with impaired *JAG*/*FIL* activity. (A–F) Cross-sections of stage 15 fruits showing the expression of the *BP::GUS* reporter in the wild type (A), *jag-1* (B), *fil-8* (C), *fil-8 jag-1* (D), *fil-8 yab3-2* (E), and *fil-8 yab3-2 jag-1* (F). (G) Relative mRNA levels of *BP* in stage 10–13 pistils quantified by qRT-PCR. r, replum; v, valve. Scale bars: 100 µm.

The phenotypic similarities of fruits in mutants affected in *JAG*/*FIL* activity genes to those of *as1* and their rescue by *bp* led us to investigate whether *BP* was also negatively regulated by *JAG*/*FIL* activity in *Arabidopsis* ovaries as it is by *AS1* [19]. Interestingly two members of this activity, the YAB1 group genes *FIL* and *YAB3*, have been previously described to repress *BP* in leaves [44]. But so far no evidence indicates that this control also occurs in fruits. We therefore analyzed the expression of the *BP::GUS* reporter construct in mutant backgrounds affected in *JAG*/*FIL* activity. In wild-type ovaries, *BP::GUS* expression is primarily detected in the medial region, corresponding to the replum (Figure 3A) [19], [43]. When the *JAG*/*FIL* activity was compromised, we observed that the intensity of the *BP::GUS* signal increased and its expression domain expanded, achieving the widest domain in the *fil yab3 jag* triple mutant (Figure 3B–3F). The exception was the *yab3* single mutant, in which the behavior of the *BP::GUS* reporter was indistinguishable from that of the wild type (data not shown). Nevertheless, we observed by qRT-PCR (quantitative real-time polymerase chain reaction) a significant increase in the expression levels of *BP* transcripts in the pistils of all backgrounds affected in *JAG*/*FIL* activity, including *yab3* (Figure 3G). Therefore, these results and those shown in the previous section indicate that the *JAG*/*FIL* activity, functioning in valves and valve margins, negatively regulates *BP* expression in medial domains and that this repression is required for the correct specification of the replum.

However, these data pose the question of whether the increased expression of *BP* in mutant backgrounds affected in *JAG*/*FIL* activity simply reflects the augmented sizes of the corresponding repla. Contrary to this line of reasoning, the GUS signal in repla of such mutants is not only wider than that of wild type but also more intense (Figure 3), suggesting that the increase in replum width is not the only cause of the higher levels of *BP* expression in mutant pistils. To further address this issue, we tested, by qRT-PCR, the expression levels of another replum gene, *RPL*, in multiple genetic conditions with impaired *JAG*/*FIL* activity and lacking *BP* function (Figure S2). In the resulting mutants, repla show reduced width as compared to the same backgrounds but with unaltered *BP* activity (Figure 2). Levels of *RPL* transcripts in wild-type and *bp* pistils were quite similar, indicating that loss of *BP* function has little effect on *RPL* expression. However, in pistils of *fil yab3 bp* and *fil jag bp*, *RPL* expression was significantly higher than in those of both the wild type and the *bp* mutant, despite the moderate width of the repla in the two triple mutants (Figure S2). Therefore, enhanced expression of *RPL*, and most likely of *BP*, in such mutant backgrounds does not exclusively depend on replum size, supporting again the negative regulation of *JAG*/*FIL* activity on replum genes.

### *BP* negatively regulates the expression of *JAG*/*FIL* activity genes

The model for mediolateral fruit patterning hypothesizes that lateral factors repress medial factors and *vice versa* [19]. Fitting with the model, we have found that *JAG*/*FIL* activity negatively regulates *BP*. Therefore we decided to study if there exists such a reciprocal repression. If that were the case, *BP* would negatively regulate *JAG*/*FIL* activity [19]. To test this prediction of the model, we made use of genetic backgrounds in which *BP* was misregulated. As *BP* is ectopically active in fruit valves of *as1* mutants [19] we therefore first examined the expression of *JAG/FIL* activity genes in *as1* pistils.

**Figure 4:**
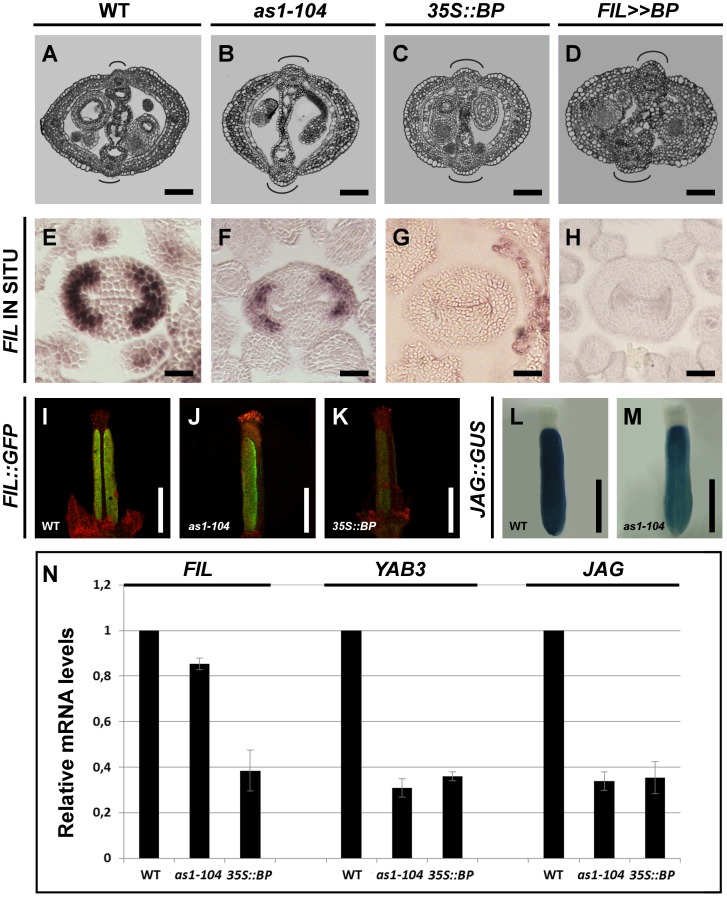
*BP* misregulation affects fruit morphology and expression of *JAG*/*FIL* activity genes. (A–D) Cross-sections of stage 15 fruits show defects in replum and valve formation in mutants misexpressing *BP*. Wild type (A), *as1-104* (B), *35S::BP* (C), and *FIL>>BP* (D). (E–K) *BP* misexpression produces a reduction in the expression of *FIL*. *In situ* hybridization of *FIL* mRNA in cross-sections of stage 8 pistils of the wild type (E), *as1-104* (F), *35S::BP* (G), and *FIL>>BP* (H); and expression of the *FIL::GFP* reporter in stage 14 gynoecia of wild type (I), *as1-104* (J), and *35S::BP* (K). Although *FIL* mRNA is undetectable by *in situ* hybridization in *35S::BP* gynoecia, the *FIL::GFP* transgene provides a more sensitive detection of *FIL* promoter expression. (L–M) Whole mount staining of anthesis gynoecia for *JAG::GUS*, showing higher expression levels in the wild-type (L) than in *as1-104* (M). (N) Relative mRNA levels of *JAG*/*FIL* activity genes in stage 10–13 pistils quantified by qRT-PCR. In A–D, the curved lines indicate replum size. Scale bars: 100 µm (A–D); 50 µm (E–H); 500 µm (I–M).

We tested by mRNA *in situ* hybridization the expression pattern of *FIL* in wild-type and *as1* gynoecia. As previously published [28], [29], [35], we found that the *FIL* mRNA is located in lateral domains of wild-type pistils (Figure 4E). However in *as1* pistils the transcript of *FIL* was detected with less intensity and in a more reduced territory (Figure 4F). This decay in *FIL* activity was also seen when the *FIL::GFP* reporter was assayed in *as1* pistils (Figure 4I, 4J and Figure S3A, S3B, S3D, S3E, S3G, S3H). A similar behavior was seen when the expression of *JAG* was monitored using a transgenic *GUS*-reporter line. In wild-type ovaries *JAG::GUS* signal is exclusively localized in lateral regions, while in *as1*, although the signal is detected in the same region, the levels of GUS activity were conspicuously lower (Figure 4L, 4M).

Interestingly, when compared to wild type, *35S::BP* plants produced flowers with fewer and narrower petals (Figure S4A, S4B), virtually phenocopying *fil* mutants (Figure S4C). These observations suggest that *FIL* activity might be severely compromised in *35S::BP* plants. We, therefore, studied the expression pattern of *FIL* in *35S::BP* pistils by *in situ* hybridization, being unable to detect any signal of *FIL* transcripts (Figure 4G). We also analyzed the *FIL::GFP* reporter in *35S::BP* gynoecia and observed a drastic reduction in GFP signal when compared to those of wild-type plants (Figure 4K and Figure S3C, S3F, S3I). Unlike the result of the *in situ* hybridization, in which no *FIL* expression was detected in *35S::BP* ovaries, the reporter provided a slight but perceptible signal, possibly because of a higher sensitivity in the detection of GFP. All together these data strongly suggest that ectopically expressed *BP*, directly or indirectly, downregulates *JAG/FIL* activity genes in ovaries. Despite this result, *35S::BP* fruits exhibited normal expression of both the *ful-1* enhancer trap (*FUL::GUS*) and the *SHP2::GUS* construct (Figure S4D, S4E)

To further investigate how ectopic *BP* expression affects the fruit, we made use of transgenic *FIL>>BP* plants, in which the *BP* coding region is transcribed in the *FIL* expression domain. For this condition, the model predicts that the expression of *BP* in lateral domains should counteract the *JAG*/*FIL* activity, affecting not only this tissue, but also the replum that would acquire a larger size. As expected, *FIL>>BP* fruits were strikingly similar to those of *35S::BP* and *as1* plants, with oversized repla and reduced valves (Figure 4A–4D). Accordingly, in *FIL>>BP* pistils, we were not able to detect *FIL* transcripts by *in situ* hybridization (Figure 4H).

Our qRT-PCR mRNA quantification also showed that in both *35S::BP* and *as1* pistils *JAG*/*FIL* activity genes were downregulated, according to the results presented above (Figure 4N). Remarkably, higher relative levels of *FIL* messenger were detected in *as1* when compared to *35S::BP*, which might be explained by the stronger expression of *BP* in *35S::BP* than in *as1* background (our unpublished results). Therefore, all the results presented so far indicate that *JAG*/*FIL* activity represses *BP*, which in turn, negatively regulates the *JAG*/*FIL* activity genes. These data further confirm that both sets of factors are mutually antagonistic in the mediolateral axis of the *Arabidopsis* fruit.

### *AS1* and *JAG* synergistically interact during mediolateral pattern formation

The strong similarities between *AS1* and *JAG*/*FIL* activity in negatively controlling *BP* expression in fruits led us to generate multiple loss-of-function mutant combinations affected in both functions to reveal the contribution of these genes to mediolateral patterning of fruits. Because of the phenotypic similarities between *as1* and plants misexpressing *BP*, we also crossed *35S::BP* plants to mutants affected in *JAG*/*FIL* activity. These sets of genetic combinations helped us to test whether the presumable fruit defects generated when *as1* and mutations in *JAG*/*FIL* activity genes combine are exclusively attributable to *BP* misexpression.

**Figure 5:**
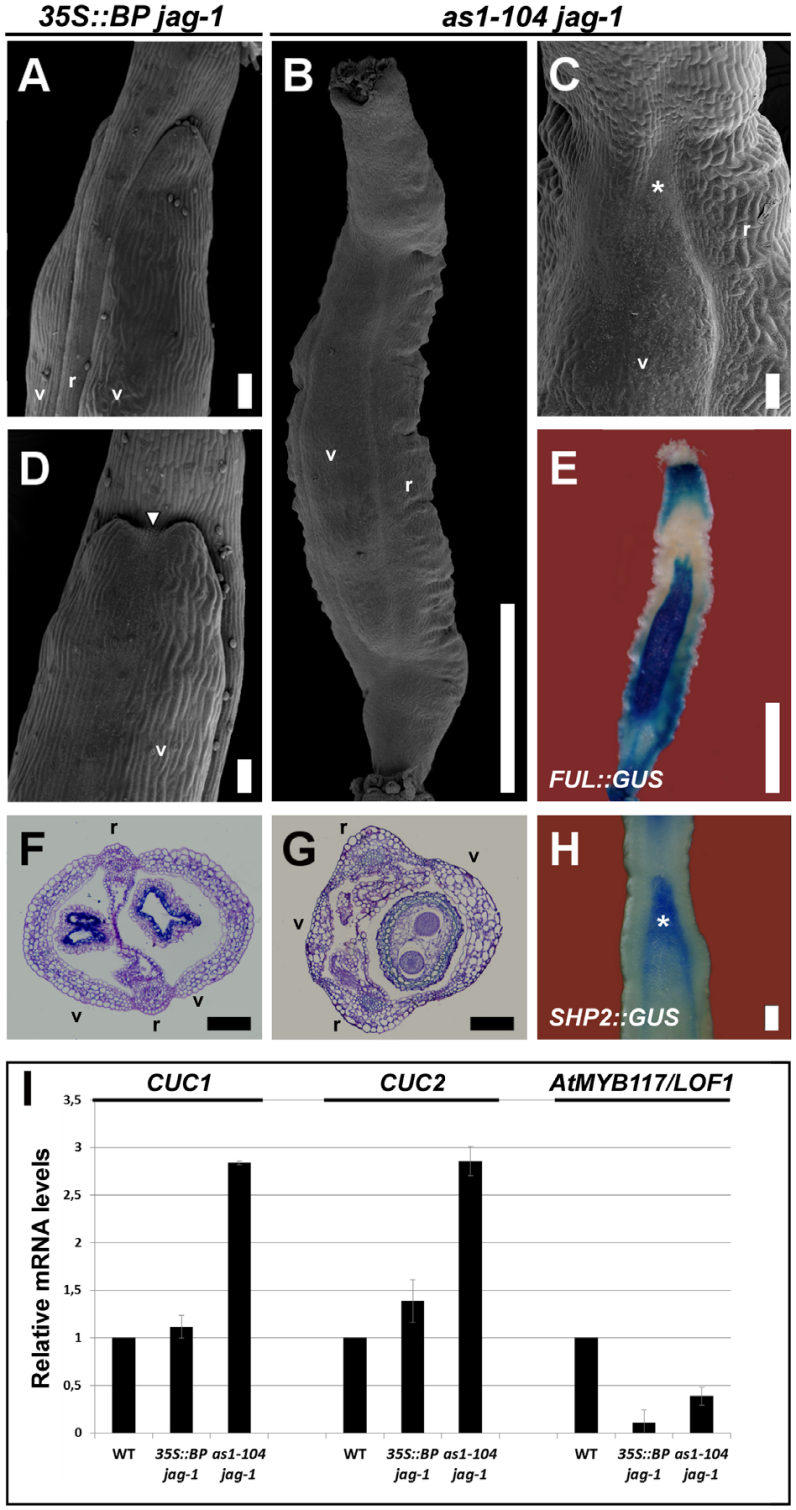
Synergistic interaction between loss-of-function alleles of *AS1* and *JAG*. (A, D) SEM micrographs and (F) cross-section of stage 17 fruits of *35S::BP jag-1* plants. These fruits show a moderate mutant phenotype (A, F) with the occasional formation of ectopic valve margin at the apical region of valves (white arrowhead in D). (B, C) SEM images and (G) cross-section of stage 17 fruits of *as1-104 jag-1* plants, showing the synergistic interaction between these two mutant alleles. Although these fruits show a similar appearance to those of *ful* mutants, with large and twisted repla and small valves (B, G), the presence of ectopic valve margin is only restricted to the apical region of the valves (asterisk in C). (E, H) Whole mount staining in stage 15 *as1-104 jag-1* fruits for *GUS* expression driven by the *ful-1* enhancer trap (E), which is detected in the small valves, and for *SHP2::GUS* (H), which is detected in the valve margin and in the apical region of valves where ectopic valve margin forms (asterisk). (I) Relative mRNA levels of *CUC1*, *CUC2* and *AtMYB117*/*LOF1* in stage 10–13 pistils quantified by qRT-PCR. r, replum; v, valve. Scale bars: 100 µm (A, C, D, F–H); 1 mm (B, E).

Fruits of *35S::BP jag* plants showed a slight increase in replum size and more reduced valves when compared to those of *35S::BP* or *jag* backgrounds (Figure 5A, 5D, 5F). As seen in *fil jag* fruits, although less frequently, we also found stripes of valve margin tissue at the upper position of the lateral-most region of *35S::BP jag* valves (Figure 5D and Figure S5A, S5E). The similarity between *35S::BP jag* and *fil jag* fruit alterations can be explained by the negative regulation of *BP* on YAB1 group genes.

Surprisingly, fruits of *as1 jag* mutants appeared by far more affected than those of *35S::BP jag* plants, showing strong reduction of valve size, as well as enlarged and twisted repla (Figure 5B, 5C, 5G; Figure S6; and Figure S7D, S7H), a phenotype reminiscent to that of *ful* mutants [10]. However, whereas *ful* valves show small and rounded epidermal cells, and do not contain any stomata, valves of *as1 jag* fruits exhibited larger cells and stomata. In line with this phenotype, the activity of *FUL::GUS* in *as1 jag* pistils was detected in the reduced valves (Figure 5E), explaining the low levels of *FUL* expression detected in this background (Figure S8).

Since in *ful* fruits the valve margin identity genes become ectopically expressed in valve tissue, we studied the activity of the *SHP2::GUS* reporter in *as1 jag* fruits. Our previous work showed that this reporter expresses normally in *as1* fruits [19]. Whole-mount staining of *as1 jag* fruits revealed normal expression for the *SHP2* reporter in the valve margin, but an expansion of the signal towards the lateral domains was detected at the upper part of the valve (asterisk in Figure 5H), consistent with an enlargement of the valve margin in this area (asterisk in Figure 5C).

The phenotypic difference between *35S::BP jag* and *as1 jag* fruits strongly suggests that, besides *BP*, *AS1* and *JAG* likely cooperate in negatively regulating other genes for mediolateral fruit patterning. It has been previously established that both *AS1* and *JAG* interact to promote sepal and petal development by downregulating the boundary-specifying genes *CUP-SHAPED COTYLEDONS1* (*CUC1*), *CUC2* and *PETAL LOSS* (*PTL*) [45]. The *LATERAL ORGAN FUSION1* (*AtMYB117*/*LOF1*) gene [46] was also considered as an additional candidate, since its ectopic expression results in enlargement of the replum [47], similar to that of *as1*, *35S::BP* or *jag* fruits. Therefore, we determined by qRT-PCR the transcript levels of these genes in wild-type, *as1 jag* and *35S::BP jag* pistils (Figure 5I). As previously reported, *PTL* is not expressed in pistil tissues [48] and basically no transcripts were detected in any of the tested backgrounds (data not shown). Transcript levels of *AtMYB117*/*LOF1* were downregulated in both *as1 jag* and *35S::BP jag* pistils (Figure 5I), which ruled out this candidate. But, interestingly, *CUC1* and *CUC2* transcripts accumulated at much higher levels in *as1 jag* than in *35S::BP jag* pistils (Figure 5I), suggesting that *CUC* genes might be involved in the strong phenotype found in *as1 jag* double mutant fruits. Interestingly, Ishida and coworkers showed that *CUC2* is involved in fruit development [49] and, in line with our hypothesis, we have observed that the *cuc2* gain-of-function allele (*cuc2-d*) [50] leads to the formation of short fruits that develop enlarged repla (Figure S9).

### Synergistic interaction between loss-of-function alleles of YAB1 group genes and *as1*

**Figure 6:**
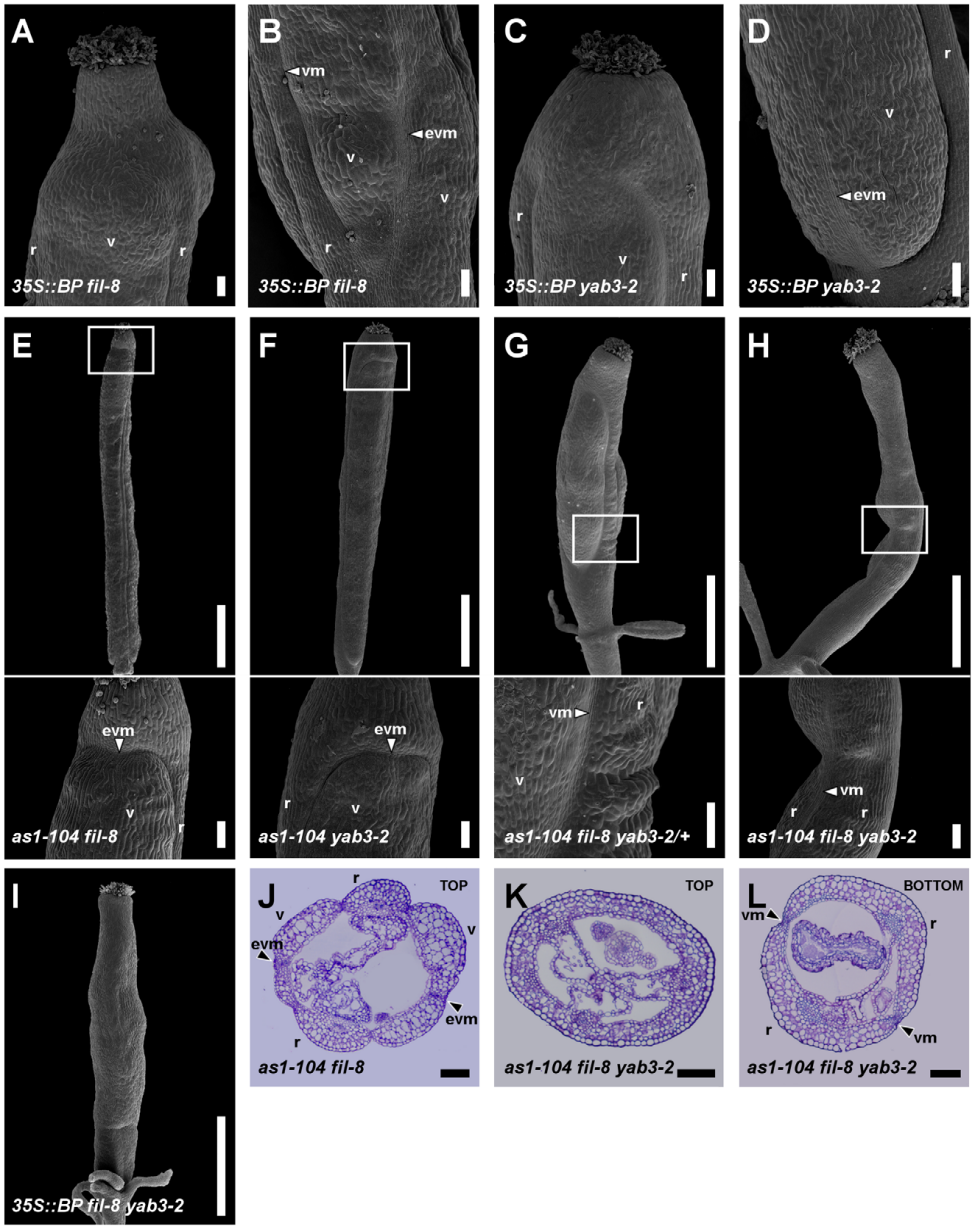
*BP* misexpression enhances the fruit defects of mutants with impaired *JAG*/*FIL* activity. (A–D) SEM micrographs of stage 17 fruits of *35S::BP fil-8* and *35S::BP yab3-2* plants. Similar to *fil yab3* fruits, in *35S::BP fil-8* (A, B) and *35S::BP yab3-2* (C, D) fruits, the apical regions lack valve margin whereas the basal regions show ectopic valve margin tissue. (E–I) SEM micrographs and (J–L) cross-sections of stage 17 fruits of several combinations of *as1-104* with mutant alleles in YAB1 group genes. In panels E–H, insets indicate the magnified area shown in the image below. The apical regions of the ovaries in *as1-104 fil-8* (E, J) and *as1-104 yab3-2* (F) fruits show ectopic valve margin, which is reminiscent of *fil YAB3*/*yab3* fruits. The increase in the mutant phenotype is evident in *as1-104 fil-8 YAB3*/*yab3-2* fruits (G), resembling *ful* mutants, although unlike these the valves of the multiple mutant show a few interspersed stomata. The fruit of the *as1-104 fil-8 yab3-2* triple mutant exhibits an extreme phenotype, which implies the complete absence of valves and the presence of two very huge repla separated by valve margin tissue in the basal region of the ovary (H, L), whereas this tissue is absent in its apical region (H, K). Fruits of *35S::BP fil-8 yab3-2* show in all their lengths the same phenotype exhibited in the apical region of triple mutant ovaries (I). evm, ectopic valve margin; r, replum; v, valve; vm, valve margin. Scale bars: 100 µm (A–D, insets in E–H, J–L,); 1 mm (upper images in E–H, I).

We next checked the effect of misregulating *BP* (*as1* and *35S::BP*) in mutant backgrounds affected in YAB1 group genes. Fruits of *35S::BP fil* and *35S::BP yab3* showed a similar phenotype, exhibiting stripes of valve margin tissue developing ectopically at the basal region of the valves, whereas the apical region of the ovary lacked valve margin (Figure 6A–6D). Because these defects were reminiscent of those seen in *fil yab3* double mutants [35] (Figure S5C, S5G), it is likely that the negative effect of *BP* on *JAG*/*FIL* activity genes could account for this phenotype. However, *as1 fil* and *as1 yab3* fruits exhibited moderate phenotypes when compared to those of *35S::BP fil* and *35S::BP yab3* (Figure 6E, 6F, 6J), but still showed a conspicuous reduction in valve size concomitant with an increase in replum width (Figure S6). In fact, in *as1 fil* mutants, the replum epidermis contained larger cells and more stomata than in any of the single mutants (Figure S7C, S7G).

In fruits of the sesquimutant *fil YAB3*/*yab3*, a stripe of valve margin tissue often appears in the apical region of the valves [35] (Figure S5B, S5F). Interestingly, we observed the formation of ectopic valve margin tissue in the valves of both *as1 fil* and *as1 yab3* fruits (Figure 6E, 6F, 6J), although with smaller size and lower frequency (40% and 30% in *as1 fil* and *as1 yab3* fruits, respectively, versus 90% in *fil YAB3*/*yab3* fruits). These observations suggest a further reduction of *JAG*/*FIL* activity in both double mutants with respect to *fil* and *yab3* single mutants.

**Figure 7:**
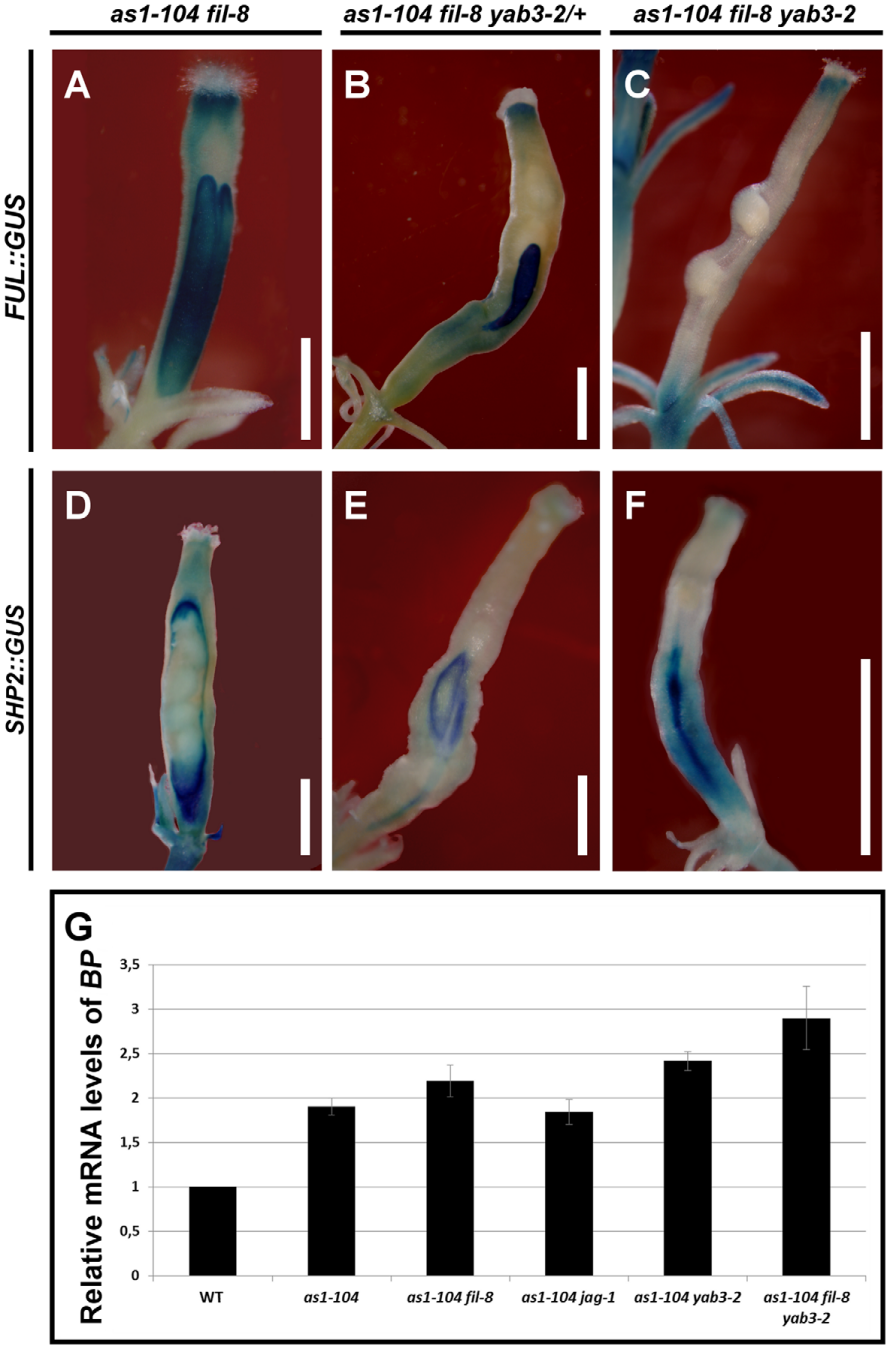
Effect of mutant combinations of loss-of-function alleles of *AS1* and YAB1 group genes on gene expression. (A–F) Whole mount staining of the *GUS* reporter under control of the *FUL* (A–C) or the *SHP2* (D–F) promoter in multiple mutant fruits carrying the *as1-104* alelle. The expression of the *ful-1* enhancer trap (*FUL::GUS*) and *SHP2::GUS* in the *as1-104 fil-8* mutant appears mostly normal, with exception of some fruits in which the expression of the *FUL::GUS* declines in both the basal and the apical regions of the ovary (A) where the *SHP2::GUS* is expressed at higher levels (D). The *as1-104 fil-8 YAB3*/*yab3-2* mutant shows reduced valves which express the *FUL::GUS* (B), whereas the *SHP2::GUS* expression is detected encompassing the small valves (E). Most *as1-104 fil-8 yab3-2* triple mutant fruits lack *FUL::GUS* in the valves (C), and exhibit a line of *SHP2::GUS* expression running along the basal two-thirds of the valve (F). (G) Relative mRNA levels of *BP* in stage 10–13 pistils quantified by qRT-PCR. Scale bars: 1 mm.

It has been previously shown that low levels of *FUL* activity in *fil YAB3*/*yab3* fruits lead to the ectopic expression of valve margin identity genes in valves [35]. Therefore, we analyzed the expression of *FUL* and the valve margin identity gene *SHP2* in *as1 fil* and *as1 yab3* pistils. By qRT-PCR assays in pistils, we found that levels of *FUL* transcripts in both double mutants were significantly reduced comparing to those in wild type or in *as1* pistils (Figure S8). In line with the phenotypes above described, *FUL::GUS* signal in *as1 fil* fruits was detected at lower levels in the apical regions of valves (Figure 7A), just where *SHP2::GUS* expresses ectopically (Figure 7D) and ectopic valve margin is produced (Figure 6E, 6J).

When one copy of *yab3* was introduced into the *as1 fil* background (*as1 fil YAB3*/*yab3* plants), the severity of the mutant phenotype was intensified and fruits exhibited smaller valves and larger repla when compared to those of *as1 fil* and *as1 yab3* double mutants (Figure 6G, Figure S6, and Figure S7E and S7I). We also noticed that *as1 fil YAB3/yab3* and *as1 jag* siliques were very similar, although in *as1 fil YAB3*/*yab3* the replum had fewer cells and was less twisted (Figure S7D, S7E, S7H, S7I). Similar to mutant fruits affected in *JAG*/*FIL* activity genes, repla of *as1 fil YAB3*/*yab3* fruits were abnormally wider, showing more and larger epidermal cells, and also presented frequent interspersed stomata, being quite difficult to distinguish them from valves (Figure S7E, S7I). In this scenario, levels of *FUL* mRNA were drastically reduced (Figure S8), and *FUL* reporter signal was restricted to small areas which correspond to the valves (Figure 7B), while the *SHP2::GUS* marked the position of the valve margins around the reduced valves (Figure 7E).

The complete loss of both *AS1* and YAB1 group genes in the *as1 fil yab3* triple mutant produced dramatic and deleterious defects on mediolateral fruit patterning (Figure 6H, 6K, 6L and Figure S6). In the basal region of *as1 fil yab3* ovaries, the most prevalent phenotype was the presence of two thin stripes of valve margin located at the lateral-most regions of the ovary, both separating what we called two giant *“super-repla”* (Figure 6H, 6L). We also found fruits with extremely small valves separated from the oversized repla by valve margin tissue (Figure S10B). The aberrant replum of *as1 fil yab3* fruits contained wide and large cells and fully developed stomata, making this tissue to adopt a similar appearance to wild-type valves (Figure S7F). In fact, the phenotype was even stronger in the apical region of the ovary where only these wide and large cells and stomata could be observed, completely lacking valve margin tissue (Figure 6H, 6K). Accordingly, *as1 fil yab3* pistils showed very low levels of *FUL* messenger (Figure S8) and *FUL* reporter activity was only detected in fruits in which valve tissue developed (Figure 7C and Figure S10A). In line with these observations, the expression of the *SHP2::GUS* reporter was mostly seen forming a stripe in the lateral-most region of *as1 fil yab3* ovaries (Figure 7F). These abnormalities make *as1 fil yab3* fruits quite different from those of *jag fil yab3* triple mutants, since the former are mainly composed of giant replum, while the latter clearly show valve and replum regions, as the signal for the *BP::GUS* revealed (Figure S11). In *35S::BP fil yab3* plants, the fruit mutant phenotype was even stronger, and both apical and basal regions of the ovary showed the same aspect as the apical region of ovaries in *as1 fil yab3* fruits (Figure 6I).

Our model predicts that an increase in the activity (or misexpression) of replum factors (*BP*) along with a reduction in the function of lateral factors (*JAG*/*FIL* activity) should lead to the formation of fruits with an enormous replum territory and very small valves [19]. The fruit phenotypes described for combinations of *as1* and mutant alleles in *JAG*/*FIL* activity genes are very much in line with these predictions (Figure 7G and Figure S6). In strong agreement, *as1 fil yab3* and *35S::BP fil yab3* plants produced fruits with huge repla that contained abnormal cell types, and an extreme reduction or abolishment of valve development (Figure 6H, 6I, 6K, 6L and Figure S10B). This phenotype is mainly due to ectopic expression of *BP* in a background with reduced *JAG*/*FIL* activity.

## Discussion

The current model for mediolateral fruit development in *Arabidopsis* hypothesizes that the final pattern is established by the concurrence of two opposing and antagonistic activities (lateral and medial factors) [7], [19]. *JAG*/*FIL* activity genes are lateral factors responsible for the establishment of the lateral pattern elements: valves and valve margins [35]. In the replum (the medial pattern element), the cooperating medial (or replum) factors *BP* and *RPL* [11], [12], [14] are required for replum formation and growth [19], [36]. The results presented in this work show that both lateral and medial factors actually repress each other, and that this mutual antagonism results in proper pattern formation along the mediolateral axis of the *Arabidopsis* fruit.

*AS1* intimately cooperates with lateral factors by negatively regulating *BP*, which prevents its ectopic expression in valves and secures correct level of *JAG*/*FIL* activity. Consequently, strong reduction of *JAG*/*FIL* activity in combination with misregulation of *BP* (by either *35S::BP* or *as1*) leads to the giant “*super-replum*” phenotype. Therefore, in this developmental program, *AS1* and its molecular partner *AS2* can be also considered as lateral factors.

### *JAG*/*FIL* activity specifies replum morphology by negatively regulating the replum factor *BP*

Besides their activities in fruit patterning, *JAG*/*FIL* activity genes have been previously described by their participation in the formation of other lateral organs. Consequently, they all are expressed in lateral organs but not in meristematic tissues. The YAB1 group genes, *FIL* and *YAB3*, promote leaf development by repressing the expression of class I KNOX meristematic genes in leaves [44], and specify ventral (abaxial) fate [28], [29], [51]. Nevertheless, although *FIL* and *YAB3* are not expressed in meristems, by means of a non-cell-autonomous mechanism, they contribute to shoot apical meristem (SAM) maintenance by negatively regulating *WUSCHEL* (*WUS*) and *CLAVATA3* (*CLV3*) genes, both expressed at the central meristem domain [52]. In fact, this mechanism also affects the floral meristem [52], and *fil yab3* mutants exhibit a high frequency of fruits with three valves (Figure 3E, 3F), possibly due to an increase in floral meristem size caused by the expansion of the *WUS* expression domain.

Similarly, the results presented in this work show that, despite *FIL* and *YAB3*, as well as *JAG*, are active in lateral regions of the ovary and not expressed in the presumptive replum (medial tissue), mutants affected in *JAG*/*FIL* activity have oversized repla with extra-large cells and interspersed stomata, indicating that these laterally expressed genes make an important contribution to the correct development of the medial region in the *Arabidopsis* fruit. Hence, it is most likely that *JAG*/*FIL* activity mediate replum development by negatively regulating, also via non-autonomous mechanisms, the expression of meristematic genes, specifically *BP*, in the replum. This is deduced 1) from the enhanced expression of *BP* in mutants affected in *JAG*/*FIL* activity genes, and 2) from the rescue of the replum phenotype in *jag bp*, *fil bp*, *fil yab3 bp* and *fil jag bp* fruits. Altogether, these data provide an additional analogy between meristem and replum, as well as between lateral organs and valves.

Nothing is known about how YAB1 group genes control *BP* expression at the molecular level, and it has been previously shown *in vitro* that FIL protein binds DNA nonspecifically [53]. In SAM homeostasis, FIL and YAB3 proteins interact with members of the LEUNIG (LUG) and SEUSS-like (SEU-like) families of transcriptional co-repressors, and the resulting multicompetent protein complexes likely recruit additional transcriptional regulators to acquire then DNA sequence specificity [54]. It is likely that a similar mechanism might be operating during mediolateral patterning of the *Arabidopsis* fruit to prevent misexpression of medial factors such as *BP*.

The *JAG* gene, similarly as *YAB3* and *FIL*, controls leaf polarity and, in cooperation with its closest paralog *NUBBIN* (*NUB*), inhibits premature tissue differentiation by maintaining cell proliferation [31], [32], [55]. Interestingly, the JAG protein contains an EAR (ERF-associated amphiphilic repression)-motif [56] near the N-terminus [31], [57]. This motif is known to be involved in transcriptional repression and critically intervenes during the molecular interaction between transcriptional regulators and co-repressors [58]–[64]. Therefore, it is possible that FIL/YAB3 and LUG/LUH (LEUNIG HOMOLOG)-SEU-like complexes might recruit JAG, and perhaps other regulatory proteins, to target specific DNA sequences. A detailed analysis of this possibility might be of interest and would corroborate, at the molecular level, the genetic interactions that occur for both SAM homeostasis and mediolateral fruit patterning.

### *AS1* cooperates with the *JAG*/*FIL* activity to repress the replum identity factor *BP*

The relationship between replum and valves closely mirrors the antagonism that there exists between meristem and lateral organs [4], [19], [20]. One of such antagonistic relationships is established between class I KNOX genes, expressed in meristem, and *AS1* expressed in leaves. In the meristem, the class I KNOX gene *STM* negatively regulates *AS1* whereas, in turn, AS1 physically interacts with AS2 to directly repress *BP* in leaves [33], [65]–[68]. Similarly, *AS1* (and *AS2*) also negatively regulates *BP* in pistils, and thus, in *as1* mutants *BP* is ectopically expressed in valves and show higher levels of expression in the replum [19].

Interestingly, *as1* and *35S::BP* pistils show similar replum defects as those described for mutants affected in *JAG*/*FIL* activity genes, in which *BP* expression is also enhanced in its own medial domain, and replum defects increase when *as1* alleles or *35S::BP* construct are combined with *jag* and/or mutant alleles in YAB1 group genes [19] (this work). These findings indicate that *JAG*/*FIL* activity and *AS* genes cooperate to repress the expression of the replum factor *BP* in the medial region of pistils, and that this regulation is critical to achieve proper replum pattern. Furthermore, we have observed that valve alterations are also drastically enhanced in these mutant combinations, and our genetic and molecular analyses also evidenced that ectopic expression of *BP* downregulates *JAG* and YAB1 group genes in lateral tissues. Therefore, we can conclude that *BP* repression in lateral regions by *AS1* (and *AS2*) plays an important role in valve development by maintaining normal levels of *JAG*/*FIL* activity.

### *AS1* and *JAG* regulate other factors besides *BP* during fruit patterning

Nevertheless, although most of the *as1* fruit phenotype can be explained by misregulation of *BP*, the lack of *AS1* does not justify all the fruit defects observed in the mutants. This is better seen in *as1 bp* fruits, which nearly had wild-type appearance but still showed some subtle abnormalities [19]. This observation indicates that, besides controlling *BP* expression, *AS1* plays additional roles in fruit.

The existence of such additional *AS1* functions is further supported by the stronger phenotype of *as1 jag* fruits when compared to those of *35S::BP jag* plants. Interestingly, *AS1* and *JAG* also interact in the flower to promote petal and sepal development by negatively regulating the boundary-specifying genes *CUC1* and *CUC2* [45]. In *as1 jag* flowers, both sepal and petal development is aborted [45]. However, in *35S::BP jag* plants these floral organs develop normally (data not shown). In pistils, our qRT-PCR data revealed that both *CUC1* and *CUC2* are upregulated in *as1 jag* at much higher levels than in *35S::BP jag*. On the other hand, *cuc2* gain-of-function allele produced an increase in replum width that resembles that of *as1* and *jag* mutants. All together these data suggest that *AS1* and *JAG* cooperate to negatively regulate *CUC* function in fruit and that this repression may play an important role in mediolateral patterning.

### Antagonistic interactions between medial and lateral factors pattern the *Arabidopsis* fruit

The basis of the model for mediolateral patterning of the *Arabidopsis* fruit lies on the antagonistic activities of medial factors (*BP* and *RPL*) and lateral factors (*JAG*/*FIL* activity genes) [19]. In accordance to the model, the giant “*super-replum*” phenotype requires both low levels of *JAG*/*FIL* activity and ectopic *BP* expression in valves. This was the case for *as1 fil yab3* or *35S::BP fil yab3* siliques (this work). On the other hand, transformation of the replum into a lateral tissue, the valve margin, requires reduction of medial factor activity and increased activity of lateral factors, as in *rpl* and *rpl bp* fruits [13], [19], [35]. All together support the idea that *BP* promotes replum fate [19], [36] and, again, strongly suggest that medial and lateral factors oppose each other to specify pattern elements along the mediolateral axis.

Pattern formation by the contribution of antagonistic activities is not uncommon in plant development. For example, leaf adaxial (dorsal)/abaxial (ventral) polarity is established by antagonistic interactions between genes that specify either abaxial or adaxial identity, such as KANADI and class III HD-Zip genes [69], [70]. During embryo development, the apical/shoot versus basal/root polarity is determined by the antagonistic relationship between class III HD-Zip and *PLETHORA* (*PLT*) genes [71].

The model also proposed that lateral and medial factors work through gradients with their minimal activities in the valve margin, where they likely overlap [19]. This easily allowed to explain the low levels of *JAG*/*FIL* activity required to produce valve margin [35]. However, recent studies have determined that *BP* is only expressed and active in the replum, so that it does not overlap in the valve margin with lateral factors [36], [43] (our unpublished results). This favours a non-overlapping model whereby the medial factors are not required to function through a gradient. Nevertheless, the low levels of *JAG*/*FIL* activity needed for promoting valve margin identity suggest a gradient in the activity of lateral factors. Above a certain threshold lateral factors specify valve fate and allow other genes to function (such as *FUL*) and below that threshold valve margin tissue forms [7], [19], [20], [35].

**Figure 8:**
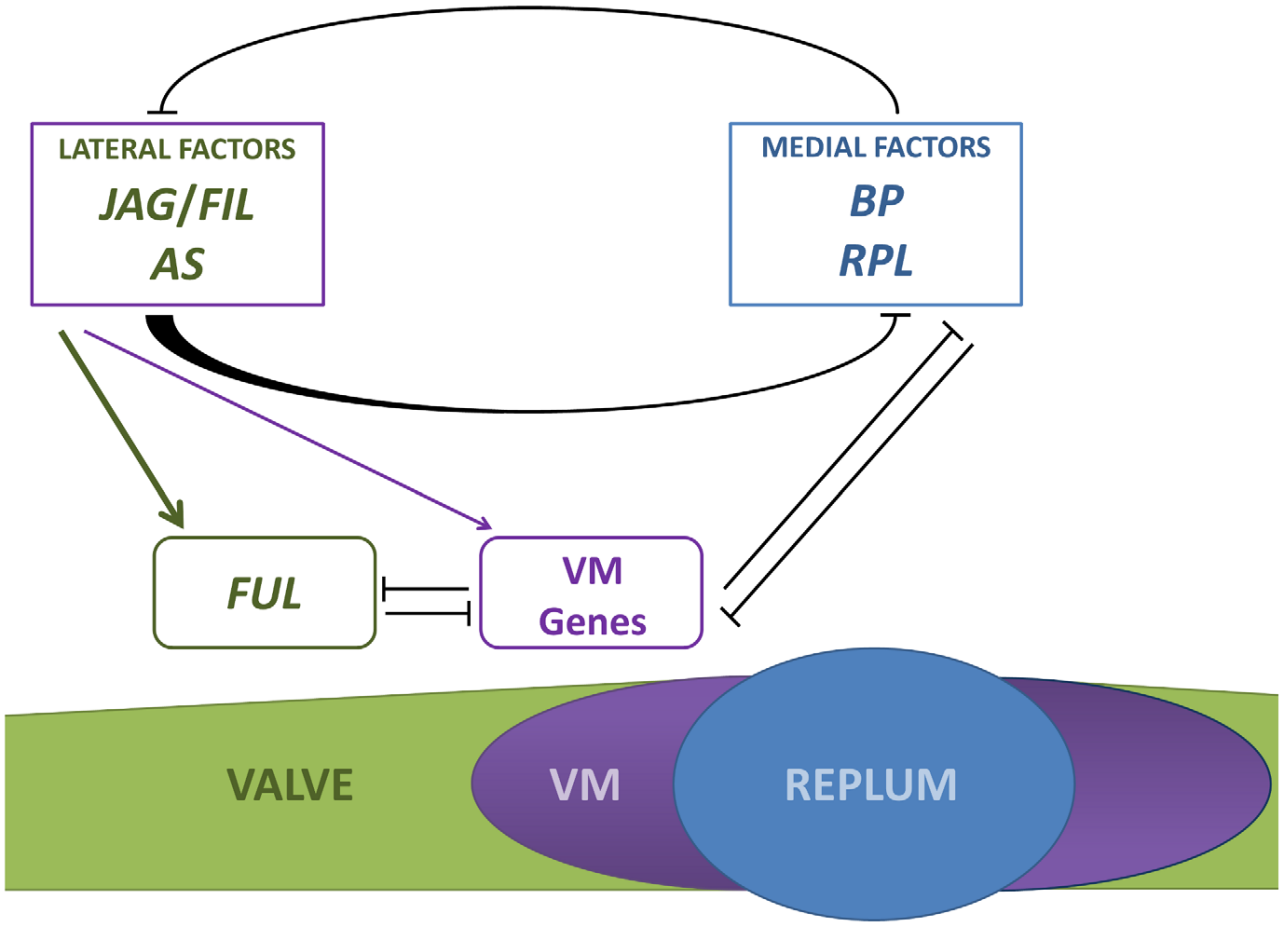
Simplified model for patterning along the mediolateral axis of the *Arabidopsis* fruit. The antagonistic activities of lateral factors (*JAG*/*FIL* activity and *AS1*/*2* genes) and medial factors (*BP* and *RPL*) are responsible for the formation and size of the three pattern elements in the mediolateral axis: valve, valve margin (VM) and replum. Lateral factors form a gradient of activity to determine valve and valve margin development. High activity of lateral factors promotes *FUL* expression in valves whereas lower levels of this activity induce the expression of valve margin identity genes (VM genes) [35]. Medial factors specify replum development [19], [36]. Lateral factors negatively regulate the expression of medial factors in a non-autonomous fashion and medial factors impede in an autonomous way the expression of lateral factors in the replum. The whole of genetic interactions along the mediolateral axis indicates that genes in all three tissue types negatively regulate the genes expressed in the other territories [13], [16], [19], [35], [36], [88].

Furthermore, the phenotypes of *as1* and *35S::BP* also support the existence of such *JAG*/*FIL* activity gradient. Misexpression of *BP* in these backgrounds reduces the expression of lateral factors, shifting to a more lateral position the region of low levels of *JAG*/*FIL* activity that produce valve margin. Farther away from the replum, these levels are high enough to activate the expression of *FUL* and specify valve development. Consequently, when mutations in *AS1*, *JAG* and YAB1 group genes combine, the more *JAG*/*FIL* activity is eliminated, the more laterally the valve margin is placed. This can be easily observed in the basal region of *as1 fil yab3* ovaries that exhibit a stripe of valve margin in their lateral-most position. In the model, *AS* function (*AS1* together with *AS2*) is integrated as another lateral factor (Figure 8).

*BP* overexpression in the repla of *ap2* mutants does not affect valve development [36], suggesting that expressions of *JAG*/*FIL* activity genes are not affected in these backgrounds and that medial factors work in a cell-autonomous way to prevent the ectopic expression of lateral factors in the presumptive replum. These observations further suggest that medial factors are not required to generate the gradient of lateral factors. On the other hand, lateral factors restrict medial factor expression to a small area that becomes the replum and, in a non-autonomous fashion, limit the expression levels of *BP* and *RPL* in medial tissues to ensure proper replum development (Figure 8). In replum tissue, *AP2* cooperates with lateral factors to negatively modulate the expression of *BP* and *RPL* in the medial domain [19], [36].

Further work will be needed to elucidate how the gradient of lateral factors is generated, although the phytohormone auxin is a possible candidate. In this sense, it has been postulated that a gradient of auxin patterns the apical-basal axis of the *Arabidopsis* fruit, with the AUXIN RESPONSE FACTOR3 (ARF3; aka ETTIN, ETT) in charge of interpreting intermediate levels of auxin to specify the ovary [72]. Interestingly, mutants affected in *JAG*/*FIL* activity genes, both with and without *as1*, show phenotypic differences along the apical-basal axis, being the phenotype always stronger in the apical region of the ovary [35] (this work), and it has been shown that *ETT* positively regulates *FIL* activity during leaf development [73], [74]. Furthermore, a recent research found that *IND* creates an auxin minimum, by regulating auxin efflux, necessary for the formation of the separation layer of the valve margin [74].

The mechanism we propose for patterning the mediolateral axis of *Arabidopsis* fruit ensures a high plasticity, and possibly may help to understand, at least in part, the variability of fruit shapes in Brassicaceae and other related species. It might be possible that subtle changes in the expression of the antagonistic factors involved in this process could produce drastic changes in the size of the different tissue types. According to this line of argument, Arnaud and coworkers have recently discovered that the reduced replum of *Brassica* plants is due to a single nucleotide change in a *cis*-regulatory element between the *RPL* orthologs of *Brassica* and *Arabidopsis*, which makes the *Brassica* wild-type allele less functional [75].

## Materials and Methods

### Plant material, growth conditions, and genetics

The mutant lines used in this work were in Landsberg *erecta* (L*er*) background and this accession was the wild-type reference. The original *35S::BP* line, in No-0 background, was introgressed four times into L*er*. In experiments involving reporter genes (*GUS* and *GFP*), the references were wild-type segregants showing the *er* phenotype, as previously described [35]. *fil-8* and *yab3-2* [44], *jag-1* [31], [35], *ful-1* [10], *bp-1* [76], *35S::BP* [22], *as1-104* [19], *FIL>>BP* [77], *SHP2::GUS* [78], *KNAT1::GUS-18* (*BP::GUS*) [65] and *FIL::GFP* [30] have been described before. *JAG::GUS* has been generated by J.R. Dinneny. Briefly, to generate the *JAG::GUS* transgenic line, a *JAG* promoter fragment was amplified from the T26J14 BAC using the primers oJD196 (5′-AAGCTTCCACTGGGCTTGTATTCCCATCC-3′) and oJD197 (5′-GGATCCAGTGGGAAATGAGAGATTGGCGTGAG-3′), which added HindIII and BamHI restriction sites to the 5′ and 3′ ends, respectively. This fragment was cloned into the pDW294 binary vector to create the construct pJD145, which was transformed after checking its integrity into Col-0 plants.

Plants were grown at 20–22°C with continuous cool-white fluorescent light as previously described [79]. Multiple mutants were identified among the F_2_ from the characteristic mutant phenotype caused by individual mutations and/or by molecular genotyping. The *fil-8*, *yab3-2* and *jag-1* alleles were genotyped using primers previously published [31], [35] (Table S1). Plants with genotypes showing defective development of stamens and poor fertility were hand-pollinated to allow the formation of fruits.

### Microscopy

Light microscopy analysis and scanning electron microscopy (SEM) were performed as previously described [79]. GFP signal was examined under a Nikon SMZ1500 stereo microscope equipped with a mercury UV lamp, and the emitted fluorescence was monitored using a filter permeable for wavelengths over 505 nm. For GUS staining, samples were treated as previously described [19], [35].

*In situ* hybridization was carried out as previously described [19] with the following modifications. The DIG-labeled antisense probe for *FIL* mRNA was obtained from the original plasmid pY1-Y (provided by J. Bowman), amplifying the insert by PCR with M13 forward and reverse primers. The amplified DNA was used as template to transcribe the probe with a T7 RNA polymerase (Fermentas).

### Quantitative real-time polymerase chain reaction (qRT–PCR)

RNA from pistils at stages 10–13 was extracted using the PureLink RNA Mini Kit (Invitrogen), and DNA contamination was removed by treatment with DNaseI (Takara). Reverse-transcription was performed from 1 µg of total RNA using the RevertAid H Minus M-MuLV Reverse Transcriptase (Fermentas). Real-time PCR was carried out using the LightCycler FastStart DNA MasterPLUS SYBR Green I (Roche) in a volume of 20 µl on the LightCycler 1.5 instrument (Roche), as previously published [80] with minor modifications. RNA levels were normalized relative to the constitutive *OTC* gene [81] and to the wild-type levels, and expression results were calculated by an efficiency correction quantification method [82]. All individual experiments were performed by triplicate, and checked twice using new cDNA every time. The reported values are averages of both biological replicates. Primers for qRT-PCR were as previously published for *AtMYB117*/*LOF1* [47], *BP* [83], *CUC1* [84], *CUC2* [85], *FUL* [86] and *OTC* [87]. A complete list of primers used in these experiments can be found in Table S1.

### Translation

A translation of the title, abstract, and author summary into Spanish is provided in Text S1.

## Supporting Information

Figure S1 Abnormal cell types in several mutant backgrounds. (A–F) SEM micrographs of stage 17 fruits showing the presence of large cells and stomata in repla of *35S::BP* (A), *as1-104* (B), *fil-8* (C), *jag-1* (D), *fil-8 yab3-2* (E) and *fil-8 jag-1* (F). Arrowheads indicate the presence of stomata. Scale bars: 100 µm.
(TIF)

Figure S2 Expression of *RPL* in mutant backgrounds lacking *BP* function. Relative mRNA levels of *RPL* in stage 10–13 pistils quantified by qRT-PCR.
(TIF)

Figure S3 Expression of *FIL::GFP* in *as1* and *35S::BP* gynoecia. (A–C) Stage 12, (D–F) stage 14 and (G–I) stage 16 gynoecia of wild type (A, D, G), *as1-104* (B, E, H) and *35S::BP* (C, F, I). Scale bars: 500 µm.
(TIF)

Figure S4 Effect of *BP* misexpresion on flowers. (A–C) Inflorescences of wild type (A), *35S::BP* (B) and *fil-8* (C). Unlike wild-type plants, *fil-8* and *35S::BP* plants show a similar flower phenotype. (D, E) Whole mount histochemical activity of *FUL::GUS* (D) and *SHP2::GUS* (E) in *35S::BP* fruits, showing basically the same pattern as in the wild type. Scale bars: 1 mm (A–C); 500 µm (D–E).
(TIF)

Figure S5 Fruit phenotypes of mutants affected in *JAG*/*FIL* activity. (A–D) SEM micrographs and (E–H) cross-sections of stage 17 fruits of several multiple mutants with impaired *JAG*/*FIL* activity. *fil-8 jag-1* fruits show ectopic valve margin at the apical region of valves (A, E). This trait is even more intense in *fil-8 YAB3*/*yab3*-2 fruits (B and F). The *fil-8 yab3-2* mutant exhibits a stronger phenotype which implies the formation of ectopic valve margin in the basal region of valves (C) and the absence of valve margin at the apical region of the ovary (C, G). The *fil-8 jag-1 YAB3*/*yab3-2* mutant shows a more severe phenotype (D, H), in which the replum in zigzag and the transformation of valve cells in valve margin cells is reminiscent of *ful* mutants. evm, ectopic valve margin; r, replum; v, valve. Scale bars: 1 mm (A–D); 100 µm (E–H).
(TIF)

Figure S6 Histograms indicating the number of outer epidermal cells in the replum and valve of mutant lines lacking *AS1* function. At least 20 repla and 20 valves were counted for each genotype. In the *as1-104 fil-8 yab3-2* triple mutant, fruits lacking valves were counted as two zeros.
(TIF)

Figure S7 Abnormal repla in mutant backgrounds lacking *AS1* function. (A–I) SEM micrographs of stage 17 fruits. *as1-104* fruits exhibit enlarged repla (A), and *as1-104 yab3-2* fruits have a similar appearance (B). This phenotype is enhanced with additional mutant alleles of *JAG*/*FIL* activity genes in *as1-104 fil-8* (C), *as1-104 jag-1* (D), *as1-104 fil-8 YAB3*/*yab3-2* (E) and *as1-104 fil-8 yab3-2* (F). Abnormal repla show the presence of large cells and stomata in *as1-104 fil-8* (G), *as1-104 jag-1* (H), *as1-104 fil-8 YAB3*/*yab3-2* (I) and *as1-104 fil-8 yab3-2* (F). Arrowheads indicate the presence of stomata. Scale bars: 100 µm.
(TIF)

Figure S8 Expression of *FUL* in mutant backgrounds lacking *AS1* function. Relative mRNA levels of *FUL* in stage 10–13 pistils quantified by qRT-PCR.
(TIF)

Figure S9 Replum phenotype in the *cuc2-d* mutant. (A–C) SEM micrographs of stage 17 fruits. Fruits of plants carrying the gain-of-function allele *cuc2-d* either in heterozygosis (B) or homozygosis (C) show oversized repla as compared to those of the wild type (A). Arrowheads indicate the positions of the valve margins. Scale bars: 100 µm.
(TIF)

Figure S10 Additional fruit phenotype in the *as1-104 fil-8 yab3-2* triple mutant. (A) Whole mount staining of *FUL::GUS* in an *as1-104 fil-8 yab3-2* fruit, showing expression in the small valve. (B) Cross-section of a stage 17 *as1-104 fil-8 yab3-2* fruit with two reduced valves. r, replum; v, valve. Scale bars: 1 mm (A); 100 µm (B).
(TIF)

Figure S11 The *fil-8 yab3-2 jag-1* triple mutant forms valve and replum territories. (A) SEM micrograph of a stage 17 fruit of the *fil-8 yab3-2 jag-1* mutant. (B) Expression of *BP::GUS* in a stage 12 *fil-8 yab3-2 jag-1* pistil indicates the presence of replum territory. Scales: 1 mm.
(TIF)

Table S1 List of oligonucleotides used in this work.
(XLS)

Text S1 Translation of the title, abstract and author summary into Spanish.
(DOC)
