# Supplementary figures and images for: Antagonistic Gene Activities Determine the Formation of Pattern Elements along the Mediolateral Axis of the Arabidopsis Fruit
Source: PLoS Genet. 2012 Nov 1;8(11):e1003020. doi: 10.1371/journal.pgen.1003020 (PMC3486860; doi:10.1371/journal.pgen.1003020)

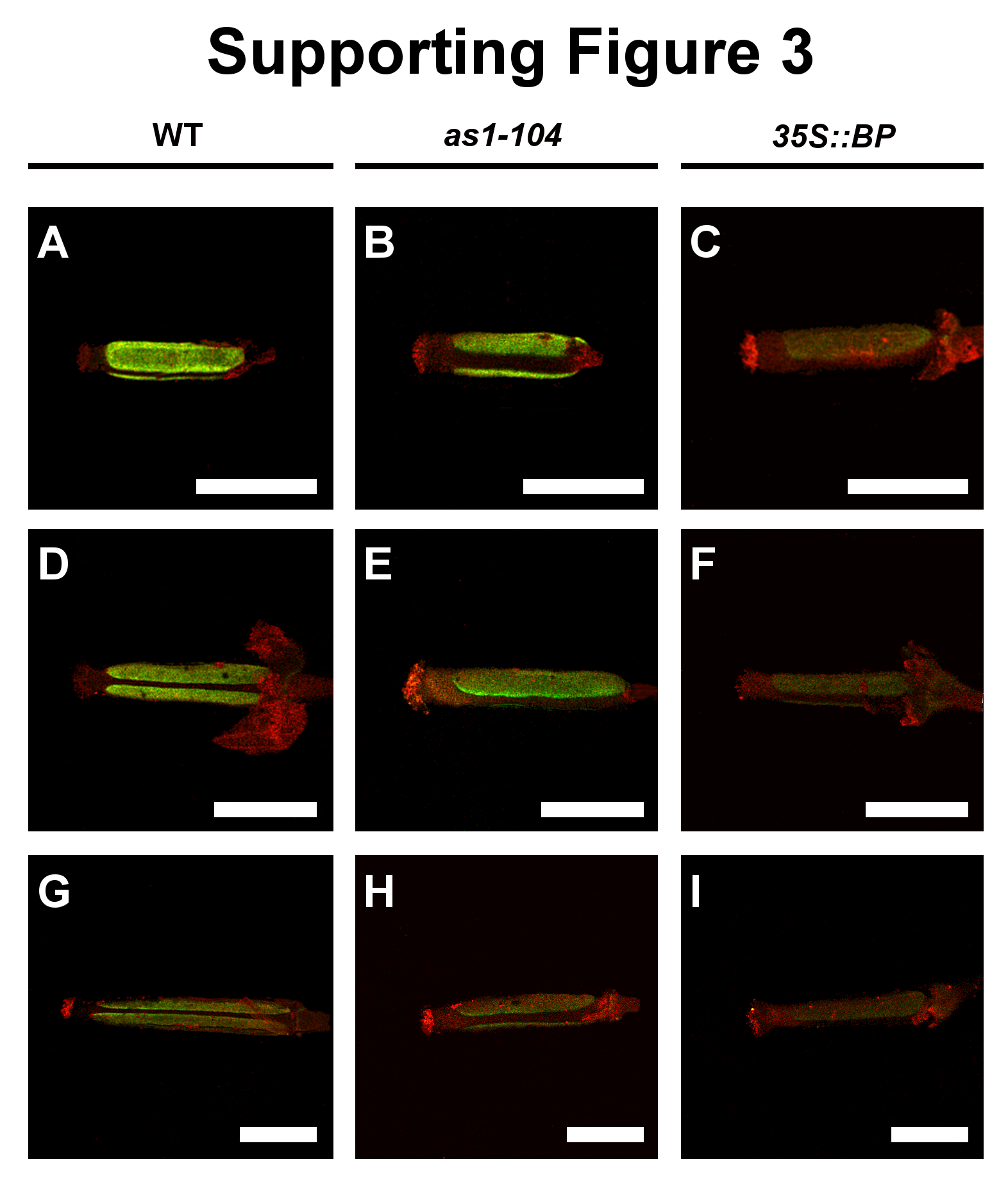

Supplement: Figure S3 — Expression of FIL::GFP in as1 and 35S::BP gynoecia. (A–C) Stage 12, (D–F) stage 14 and (G–I) stage 16 gynoecia of wild type (A, D, G), as1-104 (B, E, H) and 35S::BP (C, F, I). Scale bars: 500 µm. (TIF) [file pgen.1003020.s003.tif]

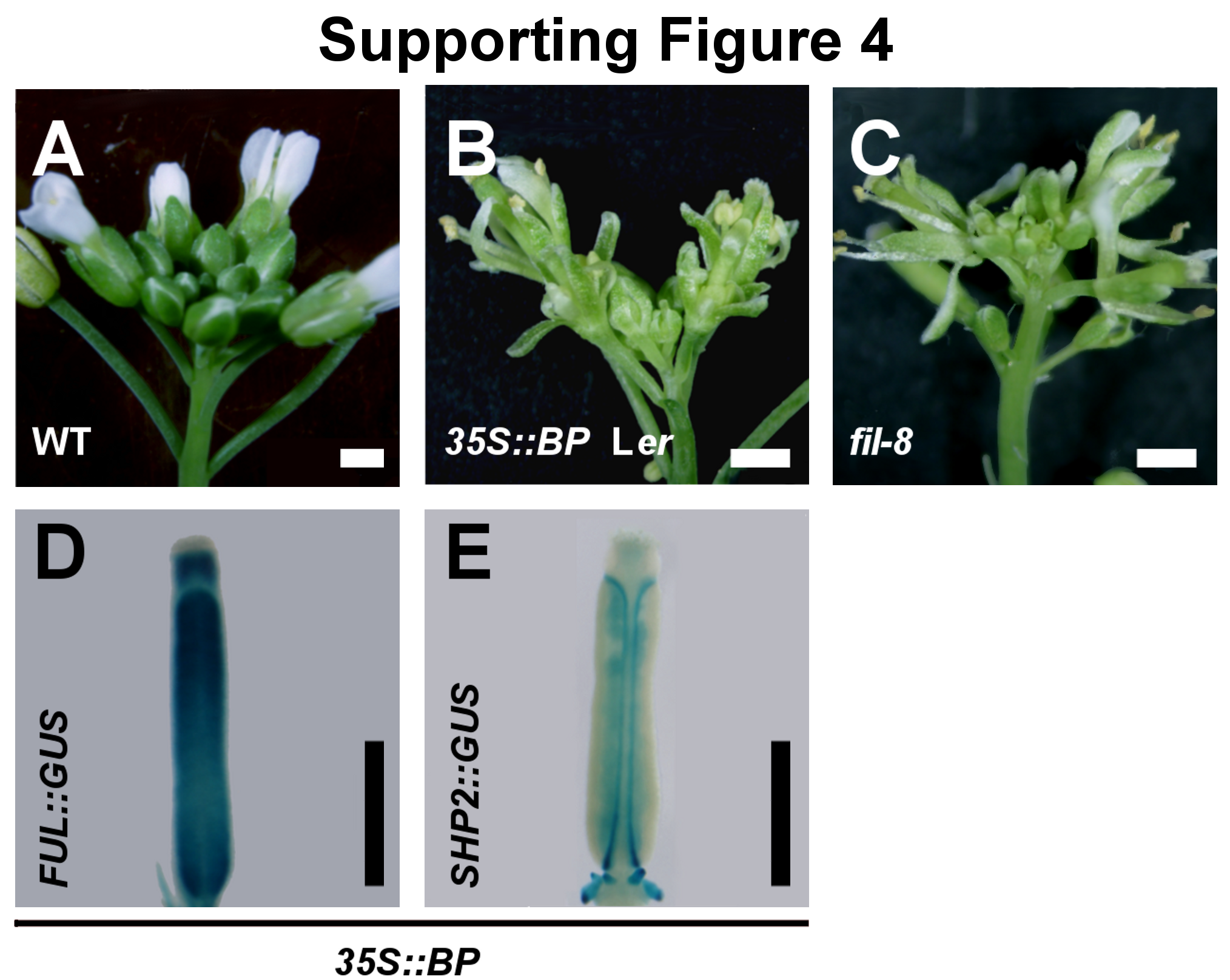

Supplement: Figure S4 — Effect of BP misexpresion on flowers. (A–C) Inflorescences of wild type (A), 35S::BP (B) and fil-8 (C). Unlike wild-type plants, fil-8 and 35S::BP plants show a similar flower phenotype. (D, E) Whole mount histochemical activity of FUL::GUS (D) and SHP2::GUS (E) in 35S::BP fruits, showing basically the same pattern as in the wild type. Scale bars: 1 mm (A–C); 500 µm (D–E). (TIF) [file pgen.1003020.s004.tif]

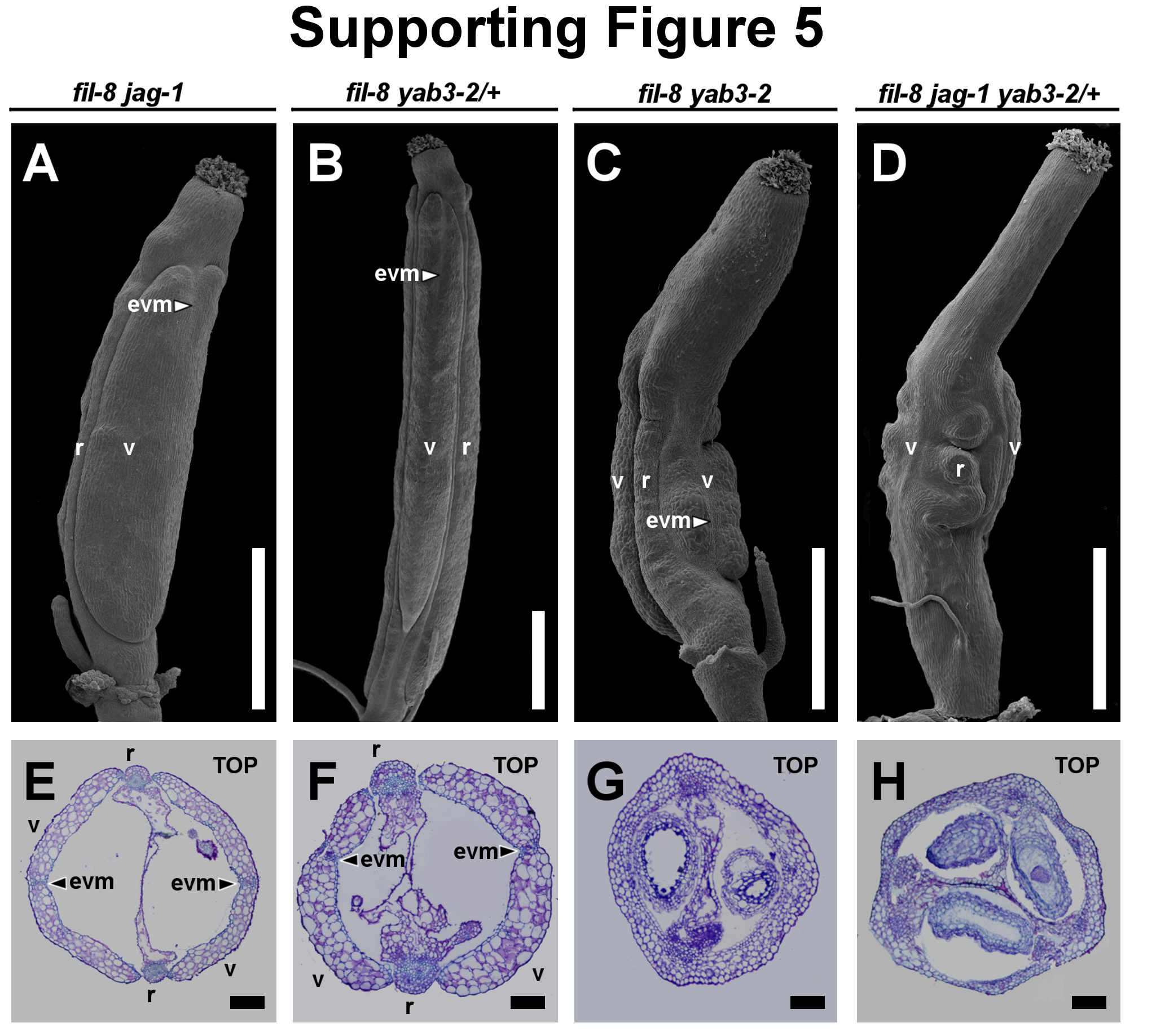

Supplement: Figure S5 — Fruit phenotypes of mutants affected in JAG/FIL activity. (A–D) SEM micrographs and (E–H) cross-sections of stage 17 fruits of several multiple mutants with impaired JAG/FIL activity. fil-8 jag-1 fruits show ectopic valve margin at the apical region of valves (A, E). This trait is even more intense in fil-8 YAB3/yab3-2 fruits (B and F). The fil-8 yab3-2 mutant exhibits a stronger phenotype which implies the formation of ectopic valve margin in the basal region of valves (C) and the absence of valve margin at the apical region of the ovary (C, G). The fil-8 jag-1 YAB3/yab3-2 mutant shows a more severe phenotype (D, H), in which the replum in zigzag and the transformation of valve cells in valve margin cells is reminiscent of ful mutants. evm, ectopic valve margin; r, replum; v, valve. Scale bars: 1 mm (A–D); 100 µm (E–H). (TIF) [file pgen.1003020.s005.tif]

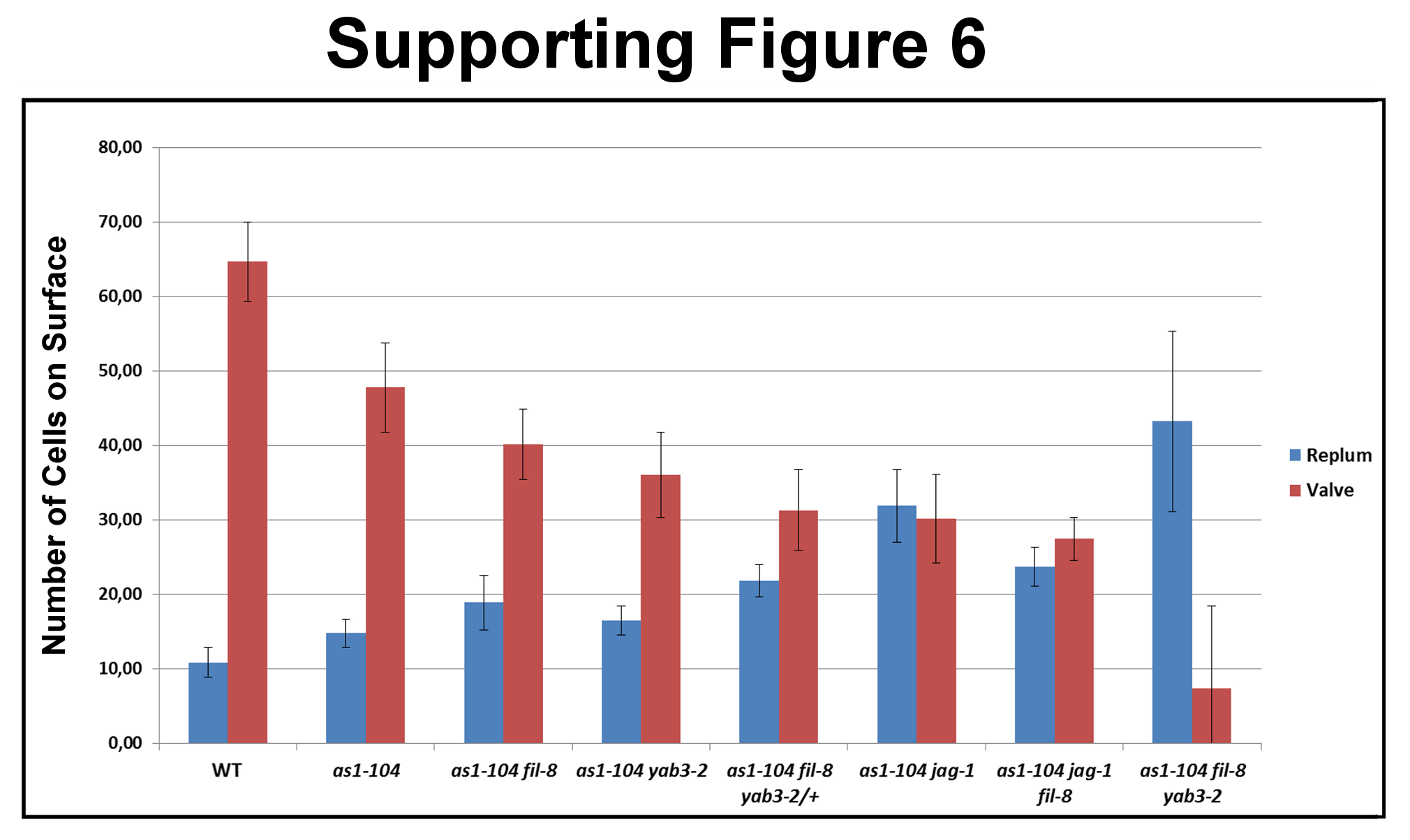

Supplement: Figure S6 — Histograms indicating the number of outer epidermal cells in the replum and valve of mutant lines lacking AS1 function. At least 20 repla and 20 valves were counted for each genotype. In the as1-104 fil-8 yab3-2 triple mutant, fruits lacking valves were counted as two zeros. (TIF) [file pgen.1003020.s006.tif]

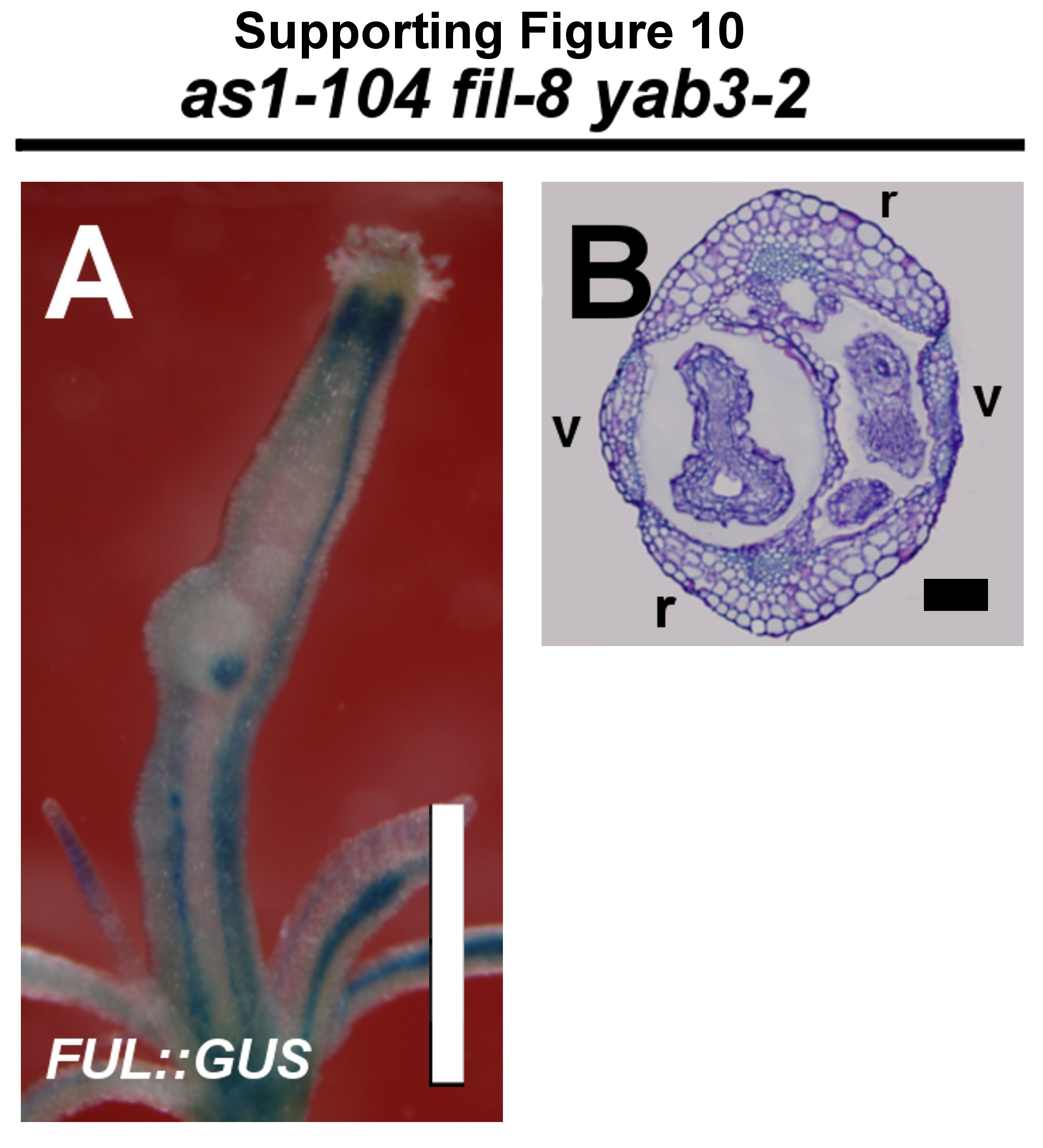

Supplement: Figure S10 — Additional fruit phenotype in the as1-104 fil-8 yab3-2 triple mutant. (A) Whole mount staining of FUL::GUS in an as1-104 fil-8 yab3-2 fruit, showing expression in the small valve. (B) Cross-section of a stage 17 as1-104 fil-8 yab3-2 fruit with two reduced valves. r, replum; v, valve. Scale bars: 1 mm (A); 100 µm (B). (TIF) [file pgen.1003020.s010.tif]

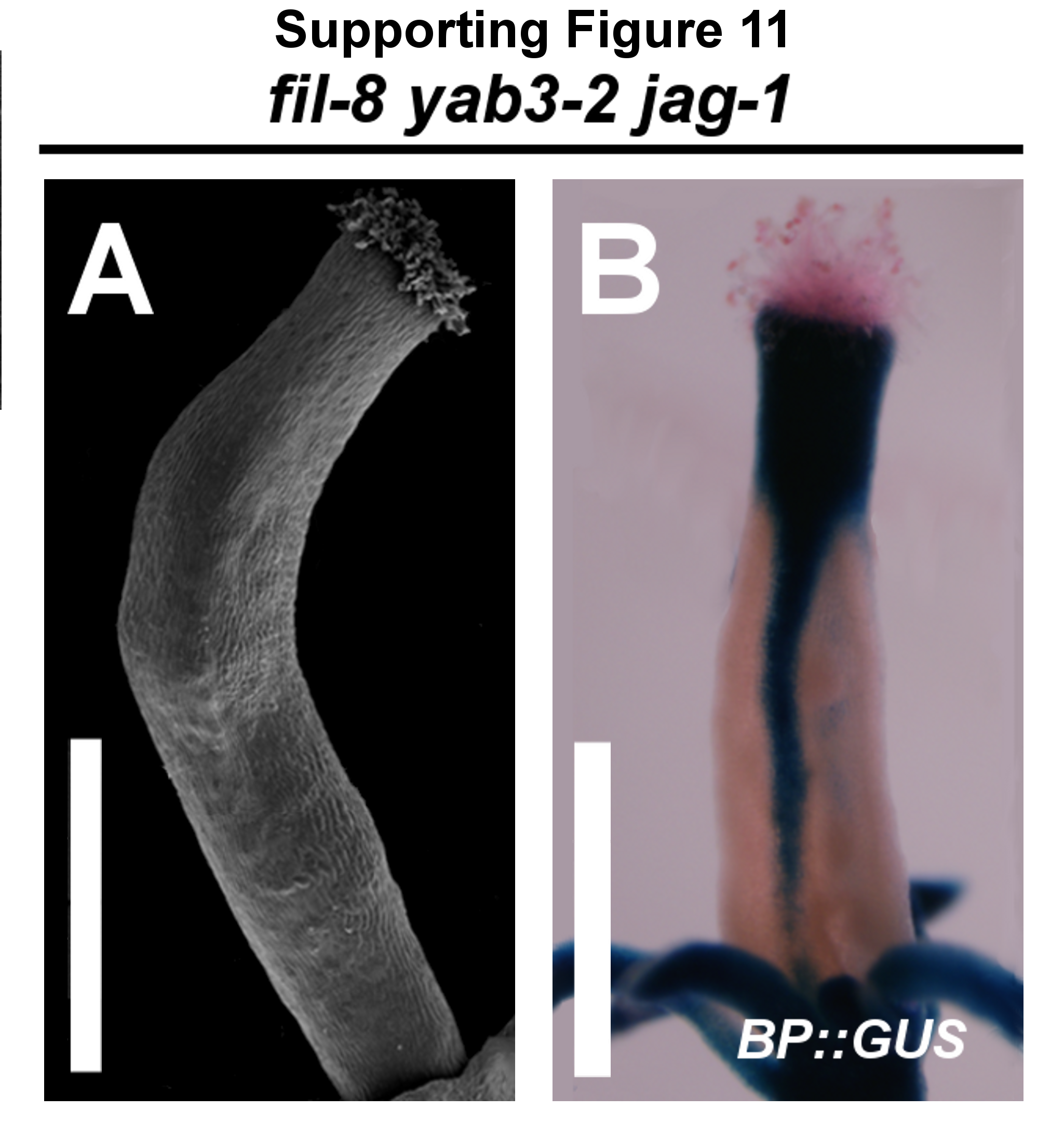

Supplement: Figure S11 — The fil-8 yab3-2 jag-1 triple mutant forms valve and replum territories. (A) SEM micrograph of a stage 17 fruit of the fil-8 yab3-2 jag-1 mutant. (B) Expression of BP::GUS in a stage 12 fil-8 yab3-2 jag-1 pistil indicates the presence of replum territory. Scales: 1 mm. (TIF) [file pgen.1003020.s011.tif]
