# Supplementary material for: Antagonistic Gene Activities Determine the Formation of Pattern Elements along the Mediolateral Axis of the Arabidopsis Fruit
Source: PLoS Genet. 2012 Nov 1;8(11):e1003020. doi: 10.1371/journal.pgen.1003020 (PMC3486860; doi:10.1371/journal.pgen.1003020)
Supplement: Text S1 — Translation of the title, abstract and author summary into Spanish. (DOC) [file pgen.1003020.s013.doc]

**Translation of the title, abstract and author summary into Spanish.**

**TÍTULO**

**Actividades génicas antagónicas determinan la formación de los elementos de patrón a lo largo del eje mediolateral del fruto de *Arabidopsis*.**

**RESUMEN**

El fruto de *Arabidopsis* consiste principalmente en un ovario maduro que muestra tres territorios bien definidos que constituyen elementos de patrón a lo largo del eje mediolateral: el replum, localizado en el plano medio de la flor, y la valva y el margen de valva, ambos de naturaleza lateral. La actividad *JAG*/*FIL*, que incluye las funciones combinadas de *JAGGED* (*JAG*), *FILAMENTOUS FLOWER* (*FIL*) y *YABBY3* (*YAB3*), contribuye a la formación de los dos elementos de patrón laterales, mientras que los genes cooperantes *BREVIPEDICELLUS* (*BP*) y *REPLUMLESS* (*RPL*) promueven el desarrollo del replum. Un modelo reciente para explicar la formación de patrón en el eje mediolateral plantea la hipótesis de que la actividad *JAG*/*FIL* y *BP*/*RPL* funcionan como factores laterales y medios, respectivamente, con características antagónicas, que tienden a reprimirse entre sí. En este trabajo, demostramos la existencia de mecanismos de exclusión mutua entre ambos tipos de factores, y cómo ello determina la formación y el tamaño de los tres territorios. Los factores medios restringen de forma autónoma a los factores laterales para que éstos se expresen únicamente fuera del replum, y los factores laterales regulan negativamente y de forma no autónoma al gen *BP*, que se expresa en la región media, asegurando así el desarrollo correcto del replum. También hemos observado que *ASYMMETRIC LEAVES1* (*AS1*), conocido por reprimir a *BP* tanto en las hojas como en los ovarios, colabora con la actividad *JAG*/*FIL*, evitando su represión por *BP* y mostrando interacciones sinérgicas con los genes de la mencionada actividad, de manera que la función génica *AS* (la función de los genes *AS1* y *AS2*, que interaccionan entre sí) se ha incorporado al modelo como un nuevo factor lateral. Nuestro modelo de factores antagónicos proporciona una explicación para los fenotipos mutantes en los frutos de *Arabidopsis*, y también puede ayudar a comprender la variación natural en la forma del fruto de las Brasicáceas y otras especies, ya que cambios sutiles en la expresión de los genes pueden dar lugar a cambios evidentes en el tamaño de los diferentes tipos de tejidos.

**RESUMEN DE LOS AUTORES**

Hay tres elementos de patrón principales en el eje mediolateral del fruto de *Arabidopsis*. Dos de ellos, las valvas y los márgenes de valvas, están situados en posiciones laterales, mientras que el tercero, llamado replum, está localizado en el plano medio de la flor. El replum expresa genes meristemáticos (factores medios) que especifican su desarrollo, mientras que la función de genes que actúan en las hojas (factores laterales) determina el desarrollo de las valvas y los márgenes de valva. En consecuencia, los elementos de patrón medios y laterales aparentemente remedan las relaciones antagónicas entre el meristemo y las hojas. De acuerdo con esto, proponemos un modelo para la formación de patrón en el eje mediolateral de los frutos, según el cual las actividades de oposición mutua de los factores medios y laterales dirigen la formación del replum, las valvas y los márgenes de valva. Concluimos que los factores medios funcionan de forma autónoma para impedir la expresión de los factores laterales en el replum, y que los factores laterales reprimen a los factores medios mediante un mecanismo no autónomo para que se produzca el desarrollo normal del replum. Nuestro modelo proporciona una explicación para los cambios en la forma del fruto en las Brasicáceas y organismos relacionados, ya sea por mutación dentro de una especie o por variación natural entre especies diferentes.
